# Insights into the Chemistry, Structure, and Biological Activity of Human Salivary MUC7 Fragments and Their Cu(II) and Zn(II) Complexes

**DOI:** 10.1021/acs.inorgchem.4c00868

**Published:** 2024-06-10

**Authors:** Klaudia Szarszoń, Silke Andrä, Tomasz Janek, Joanna Wątły

**Affiliations:** † Faculty of Chemistry, University of Wrocław, F. Joliot-Curie 14, 50-383 Wrocław, Poland; ‡ Department of Biotechnology and Food Microbiology, Wrocław University of Environmental and Life Sciences, Chełmońskiego 37, 51-630 Wrocław, Poland

## Abstract

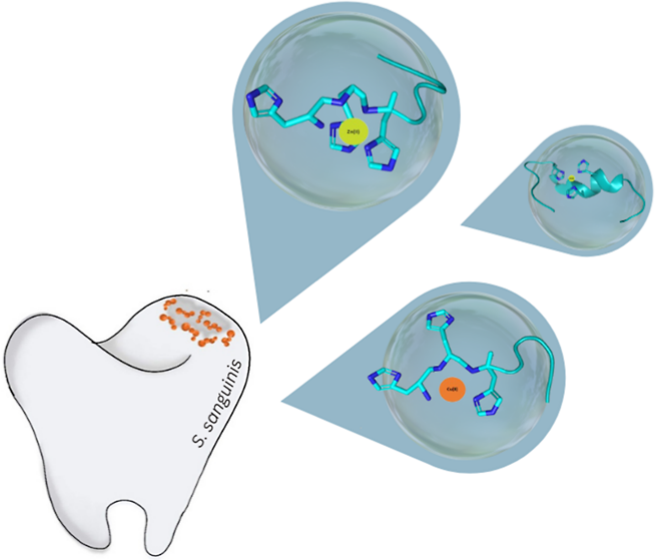

Mucin 7 (MUC7) is one of the salivary proteins whose role in the innate immune system is widely known, but still, neither its mechanism of action nor the impact of its metal coordination is fully understood. MUC7 and its fragments demonstrate potent antimicrobial activity, serving as a natural defense mechanism for organisms against pathogens. This study delves into the bioinorganic chemistry of MUC7 fragments (L1—EGRERDHELRHRRHHHQSPK; L2—EGRERDHELRHRR; L3—HHHQSPK) and their complexes with Cu(II) and Zn(II) ions. The antimicrobial characteristics of the investigated peptides and their complexes were systematically assessed against bacterial and fungal strains at pH 5.40 and pH 7.40. Our findings highlight the efficacy of these systems against *Streptococcus sanguinis*, a common oral cavity pathogen. Most interestingly, Zn(II) coordination increased (or triggered) the MUC7 antimicrobial activity, which underscores the pivotal role of metal ion coordination in governing the antimicrobial activity of human salivary MUC7 fragments against *S. sanguinis*.

## Abstract 2

Mucin 7 (MUC7), a salivary protein, exhibits antimicrobial activity against pathogens, but the mechanisms behind this remain unclear. This study explores the bioinorganic chemistry of MUC7 fragments (L1—EGRERDHELRHRRHHHQSPK; L2—EGRERDHELRHRR; L3—HHHQSPK) and their complexes with Cu(II) and Zn(II) ions. The antimicrobial efficacy of these systems against *Streptococcus sanguinis* is demonstrated, with Zn(II) coordination enhancing MUC7’s antimicrobial activity. This highlights the significance of metal ion coordination in regulating MUC7’s antimicrobial properties against *S. sanguinis*.

## Introduction

Saliva, as one of the initial defense mechanisms that organisms have developed against pathogens, serves as a crucial barrier between the underlying tissues and the external environment. Predominantly composed of inorganic components, notably water and electrolytes constituting over 99% of its composition, saliva also contains substantial concentrations of transition metal ions like zinc (13.5 ± 12.2 μg/L) and copper (1.53 ± 1.33 μg/L).^1^ The impact of these trace metals on the pathology of diverse diseases has been extensively reviewed in the literature. Furthermore, saliva encompasses a spectrum of organic compounds, including lipids, hormones, and proteins such as immunoglobulins, enzymes, histatins, and mucins.^2,3^

Mucins, serving as the primary gel-forming constituents of mucus, play a critical role in safeguarding moist epithelial surfaces across various bodily regions including the gastrointestinal tract, female genital tract, and respiratory tract. Maintaining proper regulation of mucin production is crucial for the host’s health, and ongoing research continues to enhance our understanding of the precise mechanisms by which mucins contribute to protection in the oral cavity.^4,5^ This diverse group of glycoproteins is characterized by a high degree of O-glycosylation,^6^ with the average carbohydrate content ranging from 50 to 80% by weight. Within the oral cavity, five mucins—namely, MUC5B, MUC7, MUC19, MUC1, and MUC4—were identified, each exhibiting a unique domain structure that influences their physical properties and localization. Among these, MUC7 is recognized as a fundamental component of the nonimmune host defense system, particularly within salivary mucins.^4^

Isolated from human salivary secretions, MUC7 exhibits an apparent molecular mass ranging from 150 to 200 kDa, consisting of approximately 30% protein, 68% carbohydrate, and 1.6% sulfate. Recognized as antimicrobial peptides (AMPs), MUC7 and its peptide fragments demonstrate the capacity to bind and inactivate various oral bacteria, including *Streptococcus mutans*,^7^ the periodontal pathogen *Actinobacillus actinomycetemcomitans*, *Pseudomonas aeruginosa*, and the yeast *Candida albicans*.^8^ Effective AMPs, derived from the proteolytic hydrolysis of human salivary MUC7, include 20-mer (LAHQKPFIRKSYKCLHKRCR) and 12-mer (RKSYKCLHKRCR) peptide fragments, showcasing antifungal activity and direct bactericidal effects.^9,10^ Despite the yet unknown exact mechanism underlying MUC7’s antimicrobial actions, current hypotheses suggest the potential involvement of biologically relevant metal ions in this process.^11^

Research findings indicate that the activity of antimicrobial peptides (AMPs) can be influenced by interactions with metal ions, including Zn(II) and Cu(II), thereby directly or indirectly impacting their mechanism of action. It is important to note that metal ions have the potential to alter both the net charge and the structural conformation of AMPs, leading to an enhancement of antimicrobial activity.^12,13^ Moreover, AMPs, through the binding of metal ions, restrict the pathogen access to essential metal ions vital for their life processes.^14^ This phenomenon is recognized as nutritional resistance, commonly referred to as nutritional immunity.^15,16^ Noteworthy examples of AMPs whose antimicrobial activity is closely linked to metal interactions include clavanins, pramlintide, and shepherins.^17−19^

**Figure 1:**
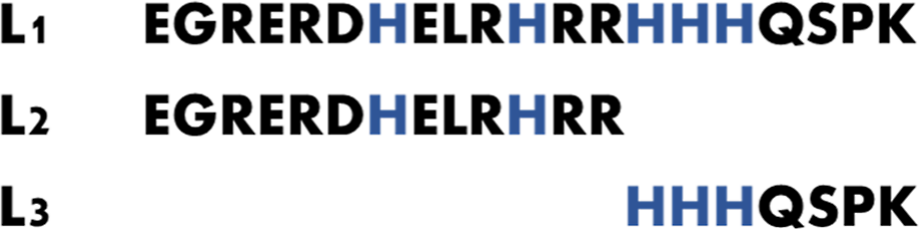
Amino acid sequence of MUC7 fragments.

We focus on three specific fragments of the human salivary protein MUC7 (Figure 1) and explore their antimicrobial activity when forming complexes with metal ions. L1 is an N-terminal fragment of human salivary MUC7.^9,20^ The peptides L2 and L3 are derived from L1 and represent fragments cleaved from MUC7 through trypsin digestion.^21^ All of them have binding sites that are very attractive for Cu(II) and Zn(II) ions: (i) His residues, (ii) amine group from the N-terminus, and (iii) amide group from the peptide bond [only in the case of Cu(II)] that exhibit a wide range of coordination modes when interacting with these metal ions.^22−24^ The different metal-binding modes are significantly influenced by (i) the quantity and placement of histidine residues in the peptide sequence and (ii) the existence of adjacent side chains that can enhance the stability of the metal-binding site through direct or indirect interactions.

The L3 peptide consisting of seven amino acid residues with a total of three histidyl residues seems to be very interesting from a coordination chemistry point of view. The sequential location of histidine residues in this peptide is quite unusual due to the presence of the HHH motif at the N-terminus, which means that two specific binding motifs are present here: (i) “albumin-like” and (ii) “histamine-like” binding sites.^25,26^ The “albumin-like” binding site, also known as the ATCUN motif [amino-terminal Cu(II) and Ni(II) binding sites], consists of a His residue at the third position in the peptide sequence and an unprotected amine group at the N-terminus. Copper(II) complexes, with a set of {1N_im_, 1NH_2_, 2N_am_} donors and a square-planar geometry (typical for the ATCUN motif), are usually observed in the pH range from 4.5 to 8.0. Cu(II) and Ni(II) complexes with this motif are highly stable.^27−30^ The second motif in this peptide, the “histamine-like” binding site, refers to the binding of Cu(II) and also Zn(II) ions by two nitrogen atoms from His-1: (i) the N-terminal amine group and (ii) the imidazole ring.^26^ Dipeptides with His as the N-terminal residue form a coordination mode similar to histamine {1NH_2_, 1N_im_}, typically in the pH range from 4.0 to 6.0.^22,31,32^ Additionally, literature data show that peptides containing a histamine-like motif can bind the Cu(II) ion more effectively than a peptide without this motif.^33^ The L1 peptide, consisting of 20 amino acid residues with a total of five histidyl residues, also contains the specific HEXXH motif. HEXXH is a typical Zn(II)-binding motif found in most zinc metalloproteinases, where two histidyl residues and glutamic acid carboxyl group coordinate a catalytic zinc ion.^34^ In the case of the L2 peptide with 13 amino acid residues, the HEXXH zinc-binding motif is also present.

In this work, we demonstrate the impact of metal ions, specifically Cu(II) and Zn(II), on the thermodynamic and structural characteristics of these peptides and explain their impact on the antimicrobial potency of these AMPs. Due to the lack of detailed coordination studies of the HHH motif at the free N-terminus, our results will be an introduction to understanding the behavior of Cu(II) and Zn(II) complexes with peptides containing this type of motif. Additionally, we propose potential mechanisms of these salivary peptides in complexes with metal ions underlying their antimicrobial effects.

## Experimental Section

### Materials

The fragments of MUC7 (EGRERDHELRHRRHHHQSPK, EGRERDHELRHRR, and HHHQSPK, all C- and N-terminally free) were purchased from Karebay Biochem (certified purity: 98%) and were used in their original state without additional purification. Cu(II) and Zn(II) perchlorate hexahydrates were highly pure products (Sigma-Aldrich). The concentrations of their stock solutions were assessed by using inductively coupled plasma spectrometry (ICP-OES). The carbonate-free stock solution of 0.1 M NaOH (Merck) was standardized potentiometrically with potassium hydrogen phthalate (Sigma-Aldrich). All samples were prepared with fresh double-distilled water. The ionic strength (*I*) was adjusted to 0.1 M by the addition of NaClO_4_ (Sigma-Aldrich). All samples were weighted using an analytical scale, Sartorius R200D.

### Mass Spectrometric Measurements

High-resolution mass spectra were acquired on an ESI-Q-TOF Maxis Impact (Bruker Daltonics) spectrometer which was used to measure Cu(II) and Zn(II) complexes (with both ligands) over a range of positive and negative values. The instrumental parameters were set as follows: scan range, *m*/*z* 150–2000; dry nitrogen gas; temperature at 170 °C; capillary voltage of 4500 V; and ion energy at 5 eV. The Cu(II) and Zn(II) complexes [(metal/ligand stoichiometry of 1:1) [ligand]_tot_ = 100 μM] were prepared in a 50:50 mixture of MeOH and H_2_O at pH 6. The samples were infused at a flow rate of 3 μL/min. Data analysis was performed by using the Bruker Compass DataAnalysis 4.0 program.

### Potentiometric Measurements

The stability constants for the proton and metal [Cu(II) and Zn(II)] complexes were determined from titration curves performed over the pH range of 2–11 at *T* = 298 K in a total volume of 2.7 cm^3^. The potentiometric titrations were carried out in 0.004 M HClO_4_ with an ionic strength of 0.1 M NaClO_4_, using a Metrohm Titrando 905 titrator equipped with a Mettler Toledo InLab Semi-Micro-combined pH electrode. The thermostabilized glass cell was equipped with a magnetic stirring system, a microburet delivery tube, and an inlet–outlet tube for argon. The solutions were titrated with 0.1 M carbonate-free NaOH. The electrodes were calibrated daily for hydrogen ion concentration by titrating HClO_4_ with NaOH using a total volume of 3.0 cm^3^. Calibration was performed under the same experimental conditions as those described above. The purity and the exact concentrations of the ligand solutions were determined using the Gran method.^35^ The ligand concentration was 0.4 mM, with a Cu(II) and Zn(II) to ligand ratio of 0.8:1. Stability constant calculations were conducted using the HYPERQUAD 2006 program.^36^ Standard deviations were computed by HYPERQUAD 2006 and were referenced to random errors exclusively. Hydrolysis constants for Cu(II) and Zn(II) ions were taken from the literature.^37,38^ The speciation and competition diagrams were computed using the HYSS program^39^ and visualized in the OriginPro 2016 program.

### Spectroscopic Studies

Absorption spectra in the UV–vis region were captured using a Jasco V-750 spectrophotometer, while circular dichroism (CD) spectra were obtained by using a Jasco J-1500 CD spectropolarimeter. The spectra were collected within the 200–800 nm range, using a quartz cuvette with an optical path length of 10 mm at *T* = 298 K in the pH range 3.0–12.0. Direct CD measurements (Θ) were transformed into mean residue molar ellipticity (Δε) using Jasco Spectra Manager. Far-UV CD spectra were recorded in the range 190–250 nm in a 0.2 mm quartz cell at *T* = 298 K for ligands and complexes at selected pH. The concentrations of solutions utilized for UV–vis and CD spectroscopic studies were consistent with those employed in the potentiometric experiments. The pH of the samples was adjusted with appropriate amounts of concentrated solutions of NaOH and HClO_4_, if necessary. Electron paramagnetic resonance (EPR) spectra were obtained at a liquid nitrogen temperature (77 K) using a Bruker ELEXSYS E500 CW-EPR spectrometer at a band frequency of 9.5 GHz. The tested ligands were prepared in aqueous solutions of HClO_4_ acid at an ionic strength of 0.1 M (NaClO_4_), and ethylene glycol (30%) was added to the solutions as a cryoprotectant. The concentration of copper ion was 0.001 M, and the metal/ligand ratio was 0.8:1. Measurements were performed within the pH range of 3.0–11.0. The experimental EPR spectra were analyzed in order to determine the EPR parameters by computer simulations using Bruker’s WIN-EPR SIMFONIA software, version 1.2 (Billerica). All obtained spectra were visualized in OriginPro 2016.

### *In Vitro* Antimicrobial Activity of Peptides and Peptide–Metal Ion Systems

The antimicrobial properties of the three peptides and their complexes were tested against human pathogenic strains. Four reference strains from American Type Culture Collection (ATCC), namely *Escherichia coli* 25922, *Pseudomonas aeruginosa* 15442, *Enterococcus faecalis* 29212, *Staphylococcus aureus* 25923, two from Polish Collection of Microorganisms (PCM), namely *Streptococcus mutans* 2502 and *Streptococcus sanguinis* 2335, and *C. albicans* SC5314 were used for antimicrobial activity assay.^40^*E. coli* ATCC 25922, *P. aeruginosa* ATCC 15422, *E. faecalis* ATCC 29212, and *S. aureus* ATCC 25923 were grown at 37 °C in Mueller–Hinton broth (MHB) (Merck Millipore, Darmstadt, Germany). *S. mutans* PMC 2502 and *S. sanguinis* PMC 2335 were cultured in Brain Heart Infusion (BHI) broth (Merck Millipore, Darmstadt, Germany) and incubated overnight anaerobically (85% N_2_, 10% H_2_, and 5% CO_2_) at 37 °C. *Candida albicans* SC5314 was grown aerobically at 37 °C on Yeast Peptone Dextrose (YPD) broth (A&A Biotechnology, Gdańsk, Poland).

### Bacterial Susceptibility Assay

The minimum inhibitory concentration (MIC) values of the peptides, their metal complexes, and metal ions were assessed using the broth microdilution method.^41^ Briefly, twofold serial dilutions of each peptide/complex in MHB, BHI, and YPD broth buffered with 10 mM MES buffer, pH 5.40 (Merck Millipore, Darmstadt, Germany) or 10 mM Hepes buffer, pH 7.4 (Merck Millipore, Darmstadt, Germany) at a volume of 100 μL were prepared in 96-well flat-bottomed microtiter plates (Sarstedt, Nümbrecht, Germany). The final concentration of each peptide/complex was ranged from 7.8 to 500 μg/mL. Likewise, the MIC test was done at a concentration of Cu and Zn ion range of 0.3 to 38 μg/mL (corresponding to the metal concentrations used in the complexes). The microtiter plate wells were inoculated with 10 μL per well of a 24 h culture of microorganisms at a final cell density of 5 × 10^6^ CFU/mL. The microplates were incubated for 24 h at 37 °C for *E. coli* ATCC 25922, *P. aeruginosa* ATCC 15422, *E. faecalis* ATCC 29212, *S. aureus* ATCC 25923, and *C. albicans* SC5314. Two oral bacteria strains, *S. mutans* PMC 2502 and *S. sanguinis* PMC 2335, were incubated at 37 °C anaerobically (85% N_2_, 10% H_2_, and 5% CO_2_), and OD_600_ was measured after 72 h using a microplate reader (Spark, Tecan Trading AG, Switzerland). The MIC end point was defined as the lowest concentration with complete (100%) growth inhibition. All assays were performed in triplicate.

## Results and Discussion

### Deprotonation Constants

A series of potentiometric titrations determined the deprotonation constants for each ligand: ten for EGRERDHELRHRRHHHQSPK (L1), seven for EGRERDHELRHRR (L2), and six for HHHQSPK (L3) (Table 1). The determined values align with those reported in the literature for similar systems.^42−44^ However, the p*K*_a_ values for the five arginine residues in L1 and L2 are outside the appropriate operating range of the electrode, making it impossible to ascertain thermodynamic parameters through potentiometric titrations.^45^ Additionally, in the case of L1 and L2 peptides, deprotonation of the C-terminal carboxyl group (and in the case of L1 also of the carboxyl group from aspartic acid) was not detected in the measurement range.^46^

**Table 1:** Deprotonation Constants (p*K*_a_) for Peptides L1, L2, and L3 and Stability Constants (Log β) for Their Complexes with Cu(II) and Zn(II) Ions in an Aqueous Solution of 4 mM HClO_4_ with *I* = 0.1 M NaClO_4_ at 298 K^a^

EGRERDHELRHRRHHHQSPK L1				EGRERDHELRHRR L2				HHHQSPK L3			
species	log β_*jk*_^b^	p*K*_a_^c^	residue	species	log β_*jk*_^b^	p*K*_a_^c^	residue	species	log β_*jk*_^b^	p*K*_a_^c^	residue
[H_11_L]^6+^	71.86(3)	3.11	Glu								
[H_10_L]^5+^	68.75(4)	3.55	Glu								
[H_9_L]^4+^	65.2(4)	4.19	Glu								
[H_8_L]^3+^	61.01(3)	5.19	His								
[H_7_L]^2+^	55.82(3)	5.87	His	[H_7_L]^2+^	35.02(1)	3.09	Asp				
[H_6_L]^+^	49.95(5)	6.22	His	[H_6_L]^+^	31.93(1)	3.42	Glu	[H_6_L]^5+^	38.07(1)	3.01	COOH
[H_5_L]	43.73(4)	6.67	His	[H_5_L]	28.51(1)	3.99	Glu	[H_5_L]^4+^	35.06(1)	4.88	His
[H_4_L]^−^	37.06(4)	7.44	His	[H_4_L]^−^	24.52(1)	4.49	Glu	[H_4_L]^3+^	30.18(1)	5.84	His
[H_3_L]^2–^	29.62(2)	9.25	H_3_N^+^	[H_3_L]^2–^	20.03(1)	5.88	His	[H_3_L]^2+^	24.34(1)	6.52	His
[H_2_L]^3–^	20.37(1)		Lys	[H_2_L]^3–^	14.15(1)	6.59	His	[H_2_L]^+^	17.82(1)	7.49	H_3_N^+^
[HL]^4–^				[HL]^4–^	7.56(1)	7.56	H_3_N ^+^	[HL]	10.33(1)	10.33	Lys

Cu(II) complexes											
EGRERDHELRHRRHHHQSPK L1				EGRERDHELRHRR L2				HHHQSPK L3			
species	log β_*jk*_^d^	p*K*_a_^e^	residue	species	log β_*jk*_^d^	p*K*_a_^e^	residue	species	log β_*jk*_^d^	p*K*_a_^e^	residue
[CuH_6_L]^3+^	56.02(2)		His								
[CuH_5_L]^2+^	51.20(2)	4.82	His								
[CuH_4_L]^+^	45.84(3)	5.36	His								
[CuH_3_L]	39.25(4)	6.59	His					[CuH_3_L]^4+^	31.81		H_3_N^+^
[CuH_2_L]^−^	32.20(3)	7.05	N_am_					[CuH_2_L]^3+^	28.62	3.19	His
[CuHL]^2–^	23.98(4)	8.22	N_am_	[CuHL]^2–^	13.2(1)		His	[CuHL]^2+^	24.12	4.50	N_am_
[CuL]^3–^	14.75(5)	9.23	H_3_N^+^	[CuL]^3–^	6.64(1)	6.56	H_3_N^+^	[CuL]^+^	18.13	5.99	His
[CuH_–1_L]^4–^	4.88(5)	9.87	N_am_	[CuH_–1_L]^4–^	–2.20(1)	8.84	N_am_	[CuH_–1_L]	10.64	7.49	N_am_
[CuH_–3_L]^6–^	–15.42(4)		Lys and Arg	[CuH_–2_L]^5–^	–11,17(1)	8.97	N_am_	[CuH_–2_L]^−^	0.32	10.32	Lys
				[CuH_–3_L]^6–^	–21.06(1)	9.89	N_am_				

Zn(II) complexes											
EGRERDHELRHRRHHHQSPK L1				EGRERDHELRHRR L2				HHHQSPK L3			
species	log β_*jk*_^d^	p*K*_a_^e^	residue	species	log β_*jk*_^d^	p*K*_a_^e^	residue	species	log β_*jk*_^d^	p*K*_a_^e^	residue
[ZnH_5_L]^2+^	48.31(2)		His								
[ZnH_4_L]^+^	42.25(2)	6.06	His								
[ZnH_3_L]	35.69(2)	6.56	His								
[ZnH_2_L]^−^	27.8(3)	7.89	H_3_N^+^								
[ZnHL]^2–^	18.93(4)	8.87	OH^–^	[ZnHL]^2–^	11.31(3)		His	[ZnHL]^2+^	15.94(2)		His/H_3_N^+^
[ZnL]^3–^	9.25(5)	9.68	OH^–^	[ZnL]^3–^	4.43(2)	6.88	H_3_N^+^	[ZnL]^+^	9.02(3)	6.92	OH^–^
[ZnH_–2_L]^5–^	–10.97(5)		Lys	[ZnH_–1_L]^4–^	–4.18(4)	8.61	OH^–^	[ZnH_–1_L]	–0.71(4)	9.73	OH^–^
								[ZnH_–2_L]^−^	–11.2(4)	10.49	Lys

a *C*_L_ = 0.4 mM; molar ratio M/L—0.8:1. The standard deviations are reported in parentheses as uncertainties on the last significant figure. The proposed coordination modes for metal complexes are described in detail in the text and are also provided in the Supporting Information [Tables S1 and S2].

b Constants are presented as cumulative log β_*jk*_ values: β(H_*j*_L_*k*_) = [H_*j*_L_*k*_]/([H]^*j*^[L]^*k*^), in which [L] is the concentration of the fully deprotonated peptide.

c p*K*_a_ values of the peptides were derived from cumulative constants: p*K*_a_ = log β(H_*j*_L_*k*_) – log β(H_*j*–1_L_*k*_).

d Cu(II) and Zn(II) stability constants are presented as cumulative log β_*ijk*_ values. L stands for a fully deprotonated peptide ligand that binds Cu(II) and Zn(II) ions: β(M_*i*_H_*j*_L_*k*_) = [M_*i*_H_*j*_L_*k*_]/([M]^*i*^[H]^*j*^[L]^*k*^), where [L] is the concentration of the fully deprotonated peptide.

e p*K*_a_ = log β(M_*i*_H_*j*_ + 1L_*k*_) – log β(M_*i*_H_*j*_L_*k*_).

### Metal–MUC7 Fragment Complexes—Characterization of Coordination Properties

To investigate the precise stoichiometry, structural, and thermodynamic properties of metal–MUC7 fragment complexes, a set of experimental methods was used: electrospray ionization mass spectrometry (ESI-MS), series of potentiometric titrations, UV–visible, circular dichroism (CD), and electron paramagnetic resonance (EPR) spectroscopies.

ESI-MS was employed to verify the purity of the investigated ligands and to determine the stoichiometries of metal binding. Investigated peptides form mononuclear complexes with Cu(II) and Zn(II) ions. No bis- or polynuclear complexes were detected using either potentiometry (Table 1) or ESI-MS (Figures S1–S6).

### Mass Spectrometry

For the Cu(II)–L1 system (Figure S1), the most intense signal corresponds to [CuL]^4+^ (*m*/*z* value at 665.81), for the Cu(II)–L2 system (Figure S3), the *m*/*z* value at 603.28 corresponds to [CuL]^3+^ and for the Cu(II)–L3 system (Figure S5), the *m*/*z* value at 485.15 corresponds to the potassium adduct of the copper complex [CuL + K]^2+^. In the case of zinc(II) complexes, the most intense signal of Zn(II)–L1 (Figure S2) corresponds to [ZnL]^3+^ (*m*/*z* value at 887.75), for the Zn(II)–L2 system (Figure S4), the *m*/*z* value at 603.28 corresponds to [ZnL]^3+^, and for the Zn(II)–L3 system (Figure S6), the *m*/*z* value at 466.68 corresponds to [ZnL]^2+^. Signals and isotopic distributions in the experimental and simulated spectra for the chosen signals are consistent and confirm the correct interpretation. Additional signals in the presented spectra are mainly potassium and sodium adducts of both ligands and complex species as well as impurities left in the measuring instrument.

### Potentiometric and Spectroscopic Studies of Cu(II) Complexes

**Figure 2:**
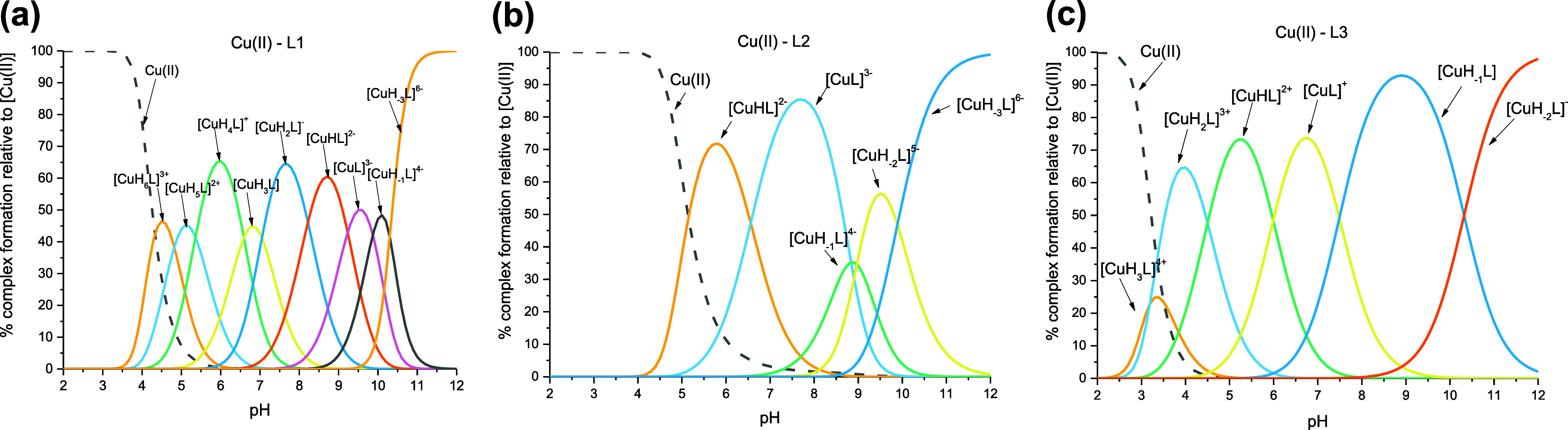
Representative distribution diagram for the (a) Cu(II)–L1 (EGRERDHELRHRRHHHQSPK), (b) Cu(II)–L2 (EGRERDHELRHRR), and (c) Cu(II)–L3 (HHHQSPK) systems in an aqueous solution of 4 mM HClO_4_, with *I* = 0.1 M NaClO_4_ dependent on pH values. *C*_L_ = 0.4 mM; molar ratio M/L—0.8:1.

Potentiometric measurements revealed the presence of nine equimolar complex species in the case of Cu(II)–L1, five in the case of the Cu(II)–L2 system, and six in the case of the Cu(II)–L3 system in the pH range of 2.50–12.00. The complex distribution diagrams and stability constant values are shown in Figure 2 and Table 1, respectively.

A careful examination of the experimental potentiometric and spectroscopic results enables a comprehensive thermodynamic and structural analysis of the complex species formation in solution, revealing the number and nature of coordinated amino acid residues and other possible groups in the peptides, as discussed in detail and presented in the description below (Table S1). The combined UV–vis and CD results allowed us to determine the binding method of Cu(II) and the geometry of these compounds formed in solution by comparing them with literature values.^47−56^

#### Cu(II)–L1 (EGRERDHELRHRRHHHQSPK) System

**Figure 3:**
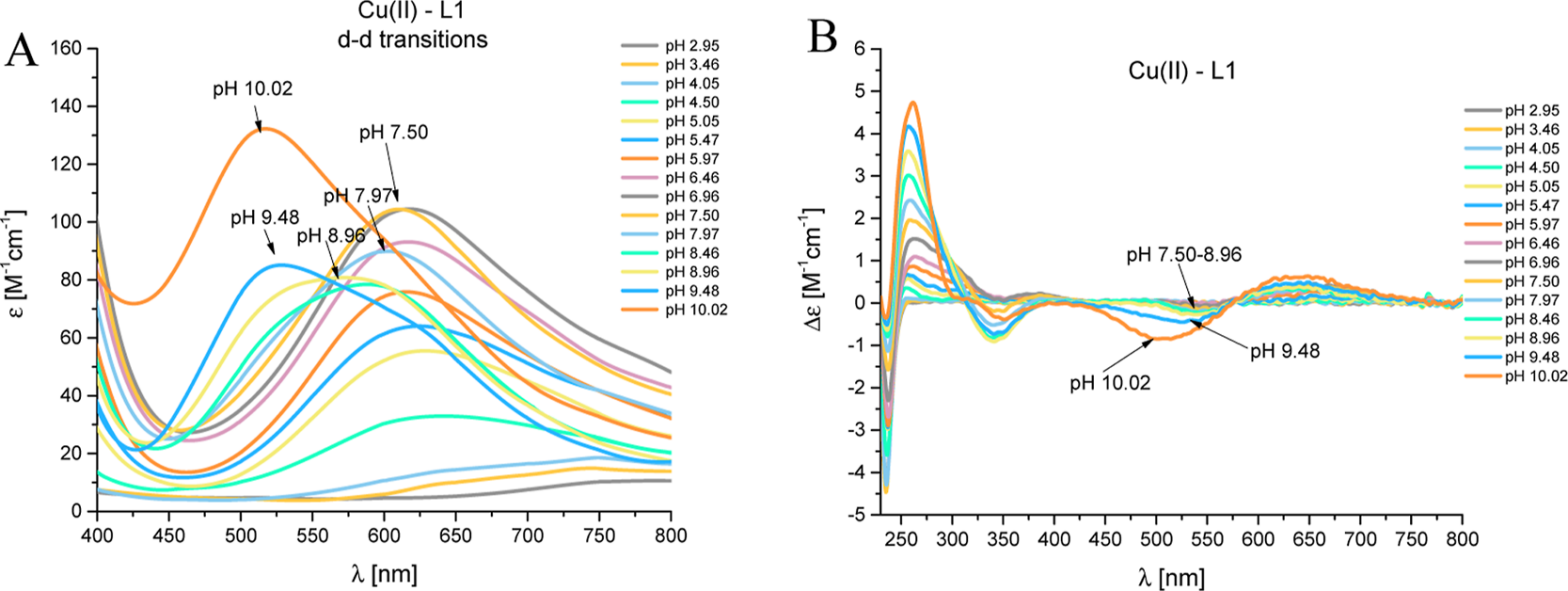
pH-dependent spectra: (A) UV–vis and (B) CD for the Cu(II)–L1 (EGRERDHELRHRRHHHQSPK) system in an aqueous solution of 4 mM HClO_4_, with *I* = 0.1 M NaClO_4_. Optical path length of 1 cm. *C*_L_ = 0.35 mM; molar ratio M/L—0.8:1.

**Figure 4:**
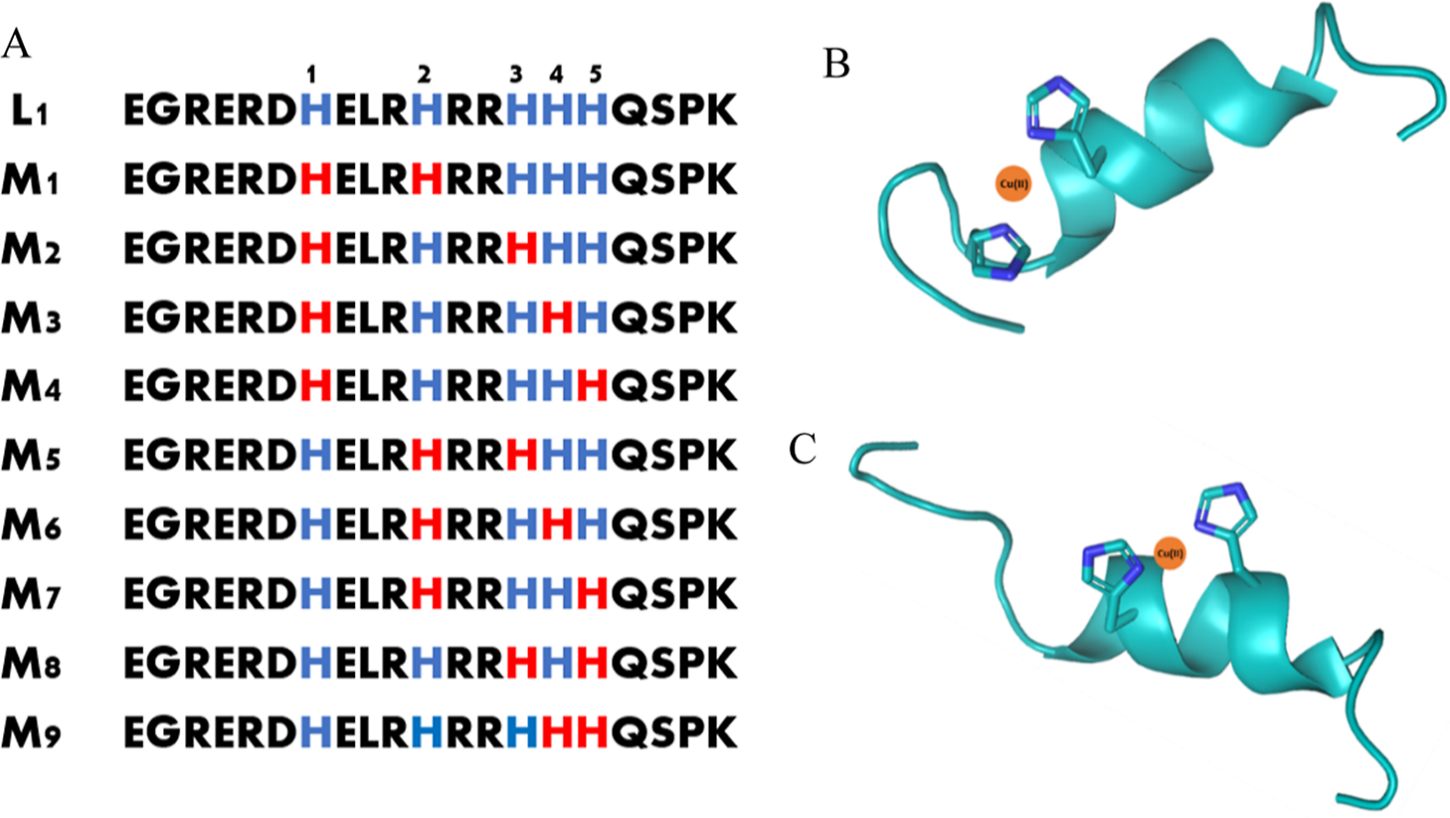
Proposed binding modes for the copper(II) complexes with L1 (EGRERDHELRHRRHHHQSPK) at pH 7.40: (A) all of the possible models (M_1_–M_9_), where two His residues binding the Cu(II) ion are marked in red; (B) schematic model for M_1_, where imidazoles from His1 and His2 can bind the Cu(II) ion; (C) schematic model for M_5_, where imidazoles from His2 and His3 can bind the Cu(II) ion. The figure was generated using PyMOL. The orange sphere represents the Cu(II) ion.

Copper(II) starts to interact with L1 around a pH as low as 4.00 (Figure 2A). The first complex species is [CuH_6_L]^3+^, with a maximum concentration at pH 4.50, where all of the carboxyl groups (from C-terminus and acidic amino acids) are already deprotonated, and most probably, Cu(II) is bound to two histidyl residues. The presence of (i) d–d transition band with a maximum absorption at 638 nm in the UV–vis spectra (Figure 3A) and (ii) a positive Cotton effect in the CD spectrum at 256 nm (Figure 3B) confirms the {2N_im_} donor set at this pH. The next complex species, [CuH_5_L]^2+^, [CuH_4_L]^+^, and [CuH_3_L], are observed due to the deprotonation of three successive His residues (p*K*_a_ = 4.82, p*K*_a_ = 5.36, and p*K*_a_ = 6.59). Lowering the p*K*_a_ values in relation to that obtained for the ligand (p*K*_a_ = 6.22, p*K*_a_ = 6.67, and p*K*_a_ = 7.44) with no significant changes in the spectroscopic data may suggest the presence of multiple complex species in equilibrium. Additionally, in this pH range, it is impossible to read the values of EPR parameters: *A*_∥_ and *g*_∥_, which further confirms this hypothesis (Figure S7). In each of these species, a maximum of two imidazole nitrogens are bound to a copper(II) ion (Figure 4). Such type of binding is referred to as the “so-called” polymorphic binding states.

The polymorphic binding mode is a situation employed by the environment to adjust the outcome of metal coordination to the prevailing physiological needs.^44^ The definition of “polymorphic binding sites” is the state in which two different sets of two His imidazole residues are bound to the metal, where one of His residues is the same in the two cases (two complex 2N in equilibrium are present). This occurrence is evident in the L1 human salivary MUC7 fragment, but it is also observed in plants’ AMPs, *e.g,.* shepherin peptides with Cu(II) ion complexes in physiological pH and animal’ systems, *e.g.*, for the Cu(II) complexes with poly-His peptides from snake venoms.^19,44,57^

The next complex species, [CuH_2_L]^−^, with a maximum concentration at pH around 7.70, most probably has a 3N coordination mode, with a {2N_im_, 1N_am_} donor set. This coordination is confirmed by the UV–vis band at 599 nm (suggesting 3N) and CD bands with (i) negative Cotton effect at 540 nm and (ii) positive Cotton effect at 651 nm (indicating the appearance of the first amide group in the coordination sphere). As the pH increases, another complex species appears, [CuHL]^2–^, which reaches its maximum concentration at pH 8.70. The broad spectroscopic band in the UV–vis spectrum (Figure 3A) suggests the equilibrium of two different coordination modes: 3N and 4N. Moreover, the overlap of the two complex species is observed in Figure 2A, which indicates a 57.5% share of [CuHL]^2–^ and 30.2% of [CuH_2_L]^−^ at this pH. Consequently, the collected data suggest the equilibrium of two complex species, first with {2N_im_, 1N_am_} and second with {2N_im_, 2N_am_} donor sets. The [CuL]^3–^ species, which reaches its maximum concentration at pH 9.50, comes from the deprotonation of the nonbonding N-terminal amine group [p*K*_a_ = 9.25 (ligand) → p*K*_a_ = 9.23 (complex)]. In the UV–vis spectrum, an apparent maximum absorption at 525 nm is observed and supports four nitrogen atoms in the coordination sphere of Cu(II) ion. Above pH 9.50, where the [CuH_–1_L]^4–^ complex species dominates, a band in the UV–vis spectrum with a clear maximum absorption at 517 nm, typical for 4N coordination, is observed. Additionally, in the CD spectrum, two positive Cotton effects are observed: at 263 and 637 nm, as well as three negative Cotton effects: at 235 nm, at 350 nm, and at 506 nm, indicating the typical square-planar geometry. These observations, together with the potentiometric data, suggest the replacement of one imidazole residue with the third amide nitrogen from the peptide bond, resulting in a {1N_im_, 3N_am_} set of donors. Above pH 11, the [CuH_–3_L]^6–^ species is observed and comes from the deprotonation of nonbonding Lys and Arg residues (for which the exact values of the deprotonation constants have not been determined potentiometrically due to the operating range of the electrode). Band maxima in the CD spectra are still at the same wavelengths, but only their intensity increases.

#### Cu(II)–L2 (EGRERDHELRHRR) System

**Figure 5:**
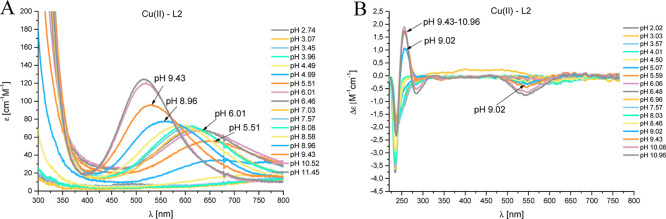
pH-dependent spectra: (A) UV–vis and (B) CD for the Cu(II)–L2 (EGRERDHELRHRR) system in an aqueous solution of 4 mM HClO_4_, with *I* = 0.1 M NaClO_4_. Optical path length of 1 cm. *C*_L_ = 0.35 mM; molar ratio M/L—0.8:1.

The first complex species, [CuHL]^2–^, appears at pH 4.00 and reaches its maximum concentration at pH 5.80 (Figure 2B). The potentiometric results and spectroscopic data: (i) the d–d band present in the UV–vis spectrum with a maximum absorbance at a wavelength of 633 nm (Figure 5A), (ii) negative Cotton effect in the CD spectrum at 239 nm (Figure 5B), and (iii) the EPR parameter values: *A*_∥_ = 180 and *g*_∥_ = 2.27 (Figure S8) indicate the {2N_im_} donor set in the case of [CuHL]^2–^. The next complex species, [CuL]^3–^, reaches the highest concentration at pH 7.70 (Figure 2B). For the formation of this complex species, the blue shift of the maximum absorption (from 633 to 609 nm, Figure 5A) is observed, which suggests the coordination of a third nitrogen atom. Moreover, the values of the EPR parameters: *A*_∥_ = 195 and *g*_∥_ = 2.22 confirm the 3N coordination mode. Based on the obtained potentiometric and spectroscopic data, a coordination mode of {2N_im_, 1NH_2_} can be suggested. As the pH increases, another complex species, [CuH_–1_L]^4–^, occurs with the maximum concentration at pH 8.90. In the UV–vis spectrum, the next shift of the maximum absorbance toward shorter wavelengths (609 → 560 nm) is observed (Figure 5A) but still indicates a 3N coordination. However, the values of EPR parameters: *A*_∥_ = 200 and *g*_∥_ = 2.20 can be assigned to both 3N and 4N coordination. At this pH, a mixture of three complex species exists in the solution (Figure 2B), and the precise characterization of [CuH_–1_L] species is nontrivial, but the appearance of a positive Cotton effect in the CD spectrum (Figure 5B) at 298 nm may suggest the coordination of the amide. Thus, for the [CuH_–1_L]^4–^ complex species, the {2N_im_, 1N_am_, and 1NH_2_} donor atom set is suggested. The next complex species, [CuH_–2_L]^5–^, observed at pH 9.50, comes from the coordination of another nitrogen atom, which is supported by the presence of (i) maximum absorbance at 531 nm in the UV–vis spectrum (Figure 5A) and (ii) EPR parameters [*A*_∥_ = 206 and *g*_∥_ = 2.19 (Figure S8)] characteristic for a 4N coordination. Based on the discussed data, for the [CuH_–2_L]^5–^ complex species, the {1N_im_, 2N_am_, and 1NH_2_} coordination mode can be suggested. For the last species, [CuH_–3_L]^6–^, reaching its maximum concentration at pH 11.90 (Figure 2B), no significant changes in the spectroscopic results were observed. Most likely, at this pH, the coordination of the third amide nitrogen atom takes place, replacing the imidazole atom or (which is not excluded) an amino group in the coordination sphere of Cu(II). Thus, for the [CuH_–3_L]^6–^ complex species, two sets of donor atoms can be suggested: {3N_am_, 1NH_2_} or {3N_am_, 1N_im_}.

#### Cu(II)–L3 (HHHQSPK) System

**Figure 6:**
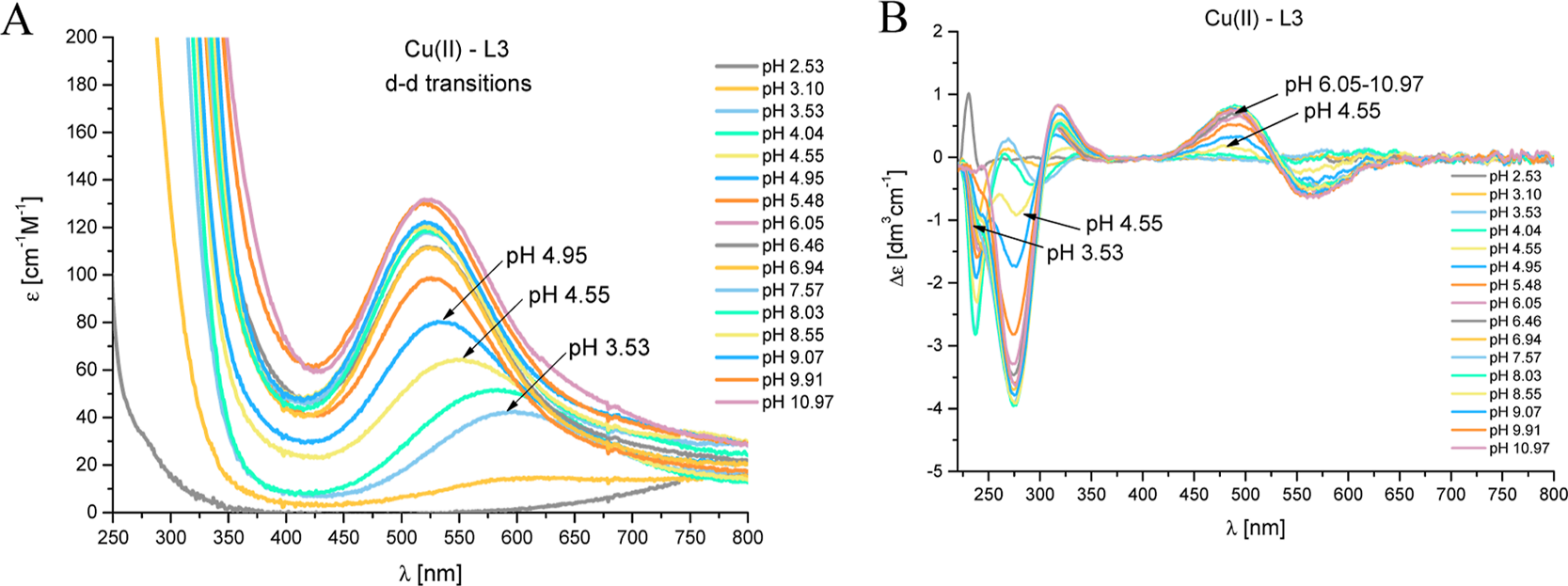
pH-dependent spectra: (A) UV–vis and (B) CD for the Cu(II)–L3 (HHHQSPK) system in an aqueous solution of 4 mM HClO_4_, with *I* = 0.1 M NaClO_4_. Optical path length of 1 cm. *C*_L_ = 0.35 mM; molar ratio M/L—0.8:1.

The first complex species detected in this system at low pH, [CuH_3_L]^4+^ (Figure 2C), starts to form at pH 2.50 and reaches its maximum concentration at pH 3.40. This complex species almost completely coincides with the [CuH_2_L]^3+^ species in the complex distribution diagram (Figure 2C). The d–d band in the UV–vis spectrum (Figure 6A) with a maximum absorption at 600 nm suggests coordination of two nitrogen atoms. Moreover, based on the potentiometric data and the occurrence of three Cotton effects in the CD spectrum: (i) negative at 236 nm, (ii) positive at 269 nm, and (iii) positive at 617 nm (Figure 6B), the most probable donors in the case of [CuH_3_L]^4+^ complex species are the N-terminal amine group and imidazole nitrogen from the histidine at the first position in the peptide sequence (“histamine-like” coordination mode).^26,58^ For the next complex species, [CuH_2_L]^3+^ with a maximum concentration at pH 4.00, a blue shift (600 → 583 nm) in the UV–vis spectrum is observed, suggesting a 3N coordination mode. Most likely, the next His residue binds the Cu(II) ion at this pH (significant lowering of the p*K*_a_ value in the complex to the corresponding p*K*_a_ value in the ligand: 3.19 → 5.84), resulting in the {2N_im_, 1NH_2_} donor set. At pH 5.30, where the [CuHL]^2+^ complex species dominate (Figure 2C), a clear shift of the absorption maximum toward shorter wavelengths (from 583 to 528 nm, Figure 6A) in the UV–vis spectra is observed, suggesting a 4N coordination mode. At the same time, new bands appear in the CD spectrum: with (i) positive Cotton effects at 314.8 and 485 nm and (ii) a negative Cotton effect at 571 nm (Figure 6B), which indicate the coordination of amide nitrogen. Based on the above results, the suggested donor set for the [CuHL]^2+^ complex species is {2N_im_, 1NH_2_, 1N_am_}. When the pH increases, [CuL]^+^ complex species with the maximum concentration at pH 6.70 is observed. This species is formed as a result of deprotonation of the last His residue, which is not involved in the Cu(II)-ion binding (comparable p*K*_a_ values in the complex and ligand: 5.99 and 6.52, respectively), and no changes are observed in the UV–vis and CD spectra. The formation of the next complex species [CuH_–1_L] (the most dominant in the pH range from 7.50 to 10.30) is accompanied by a slight increase in the intensity of the bands in the visible region in the CD and UV–vis spectra (Figure 6). The most probable in this case comes to the replacement of one of the imidazole nitrogens with an amide nitrogen atom, resulting in a {1N_im_, 1NH_2_, 2N_am_} set of donors, characteristic for “albumin-like” binding *via* the ATCUN motif. The coordination mode for the next complex species, [CuH_–2_L]^−^, remains unchanged (the band maxima in the spectroscopic spectra are still at the same wavelengths). The p*K*_a_ value assigned to this complex species comes from the deprotonation of the nonbinding Lys residue with p*K*_a_ = 10.33.

**Figure 7:**
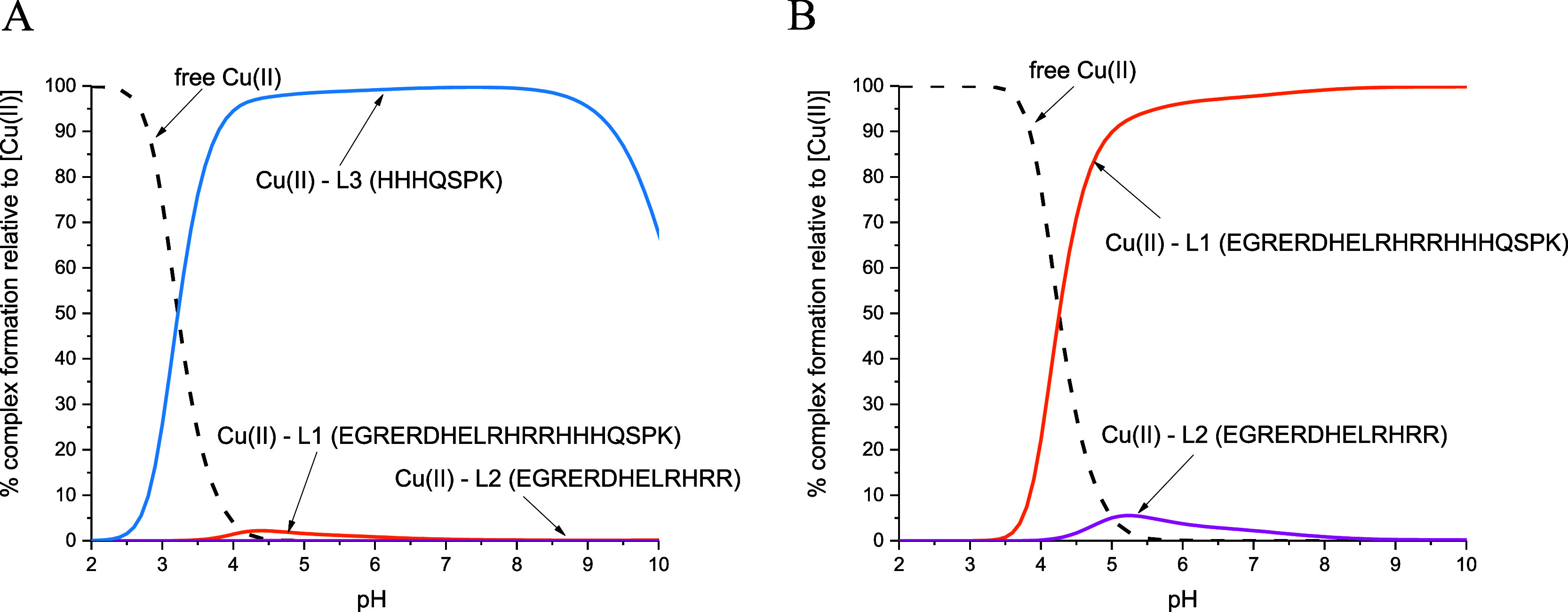
Competition plot for Cu(II) complexes with MUC7 fragments: (A) L1—EGRERDHELRHRRHHHQSPK (orange), L2—EGRERDHELRHRR (pink), and L3—HHHQSPK (blue) and (B) L1—EGRERDHELRHRRHHHQSPK (orange) and L2—EGRERDHELRHRR (pink) systems based on potentiometric data (Table 1), describing complex formation at different pH values in a hypothetical situation, in which equimolar amounts of all the reagents are mixed. Conditions: *T* = 298 K, [Cu(II)] = [L1] = [L2] = [L3] = 0.001 M.

To compare the binding efficiency of Cu(II) ions by the studied MUC7 fragments, competition plots were prepared (Figure 7). As shown in Figure 7A, L3 (HHHQSPK) is the ligand that most effectively binds Cu(II) ions over the entire pH range, most likely due to the presence of “histamine-like” and “albumin-like” binding sites in the sequence. L1 (EGRERDHELRHRRHHHQSPK) and L2 (EGRERDHELRHRR) peptides bind copper(II) ions with a much lower efficiency compared to L3, where L2 is the least effective of them. Peptides containing the ATCUN motif are very well known from forming the most thermodynamically stable complexes with Cu(II) ions.^28−30^ In order to accurately assess the stability of Cu(II) complexes with L1 and L2 (both showing an apparently similar affinity for copper ions in Figure 7A), an additional competition plot was generated (Figure 7B), which shows the big difference between them. There is one probable factor contributing to this significant disparity—the presence of polymorphic binding sites in the case of the Cu(II)–L1 system.

### Potentiometric Studies of Zn(II) Complexes

**Figure 8:**
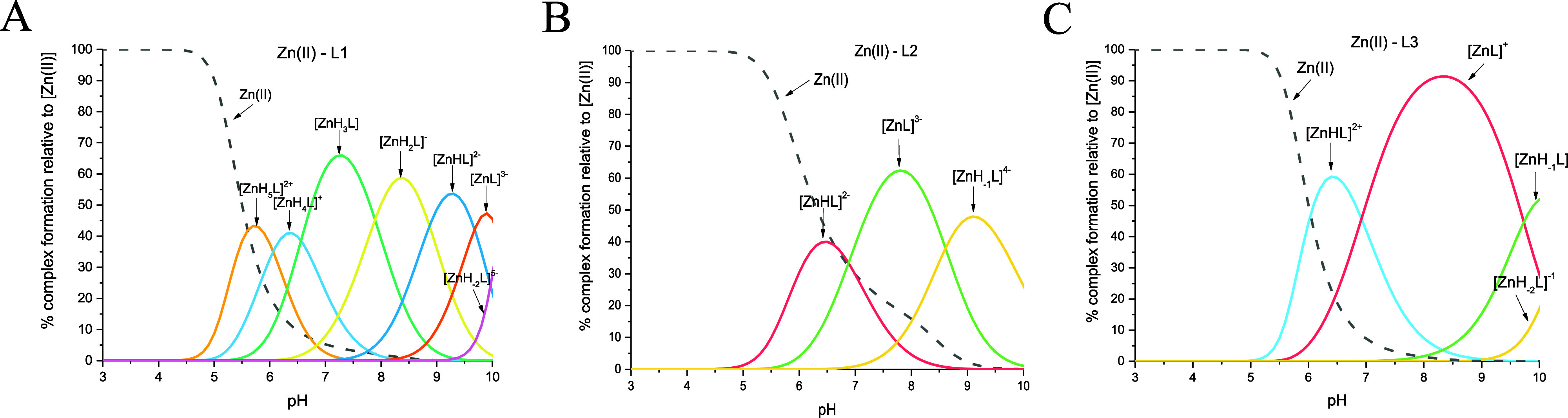
Representative distribution diagram for the (A) Zn(II)–L1 (EGRERDHELRHRRHHHQSPK), (B) Zn(II)–L2 (EGRERDHELRHRR), and (C) Zn(II)–L3 (HHHQSPK) systems in an aqueous solution of 4 mM HClO_4_, with *I* = 0.1 M NaClO_4_ dependent on pH values. *C*_L_ = 0.4 mM; molar ratio M/L—0.8:1.

The titration curves for the Zn(II)–MUC7 fragment systems (Figure 8) were fitted best by assuming the formation of seven complex species in the case of L1 (EGRERDHELRHRRHHHQSPK), three species in the case of L2 (EGRERDHELRHRR), and four species in the case of L3 (HHHQSPK). The results from the potentiometric titrations are summarized in Tables 1 and S2.

#### Zn(II)–L1 (EGRERDHELRHRRHHHQSPK) System

**Figure 9:**
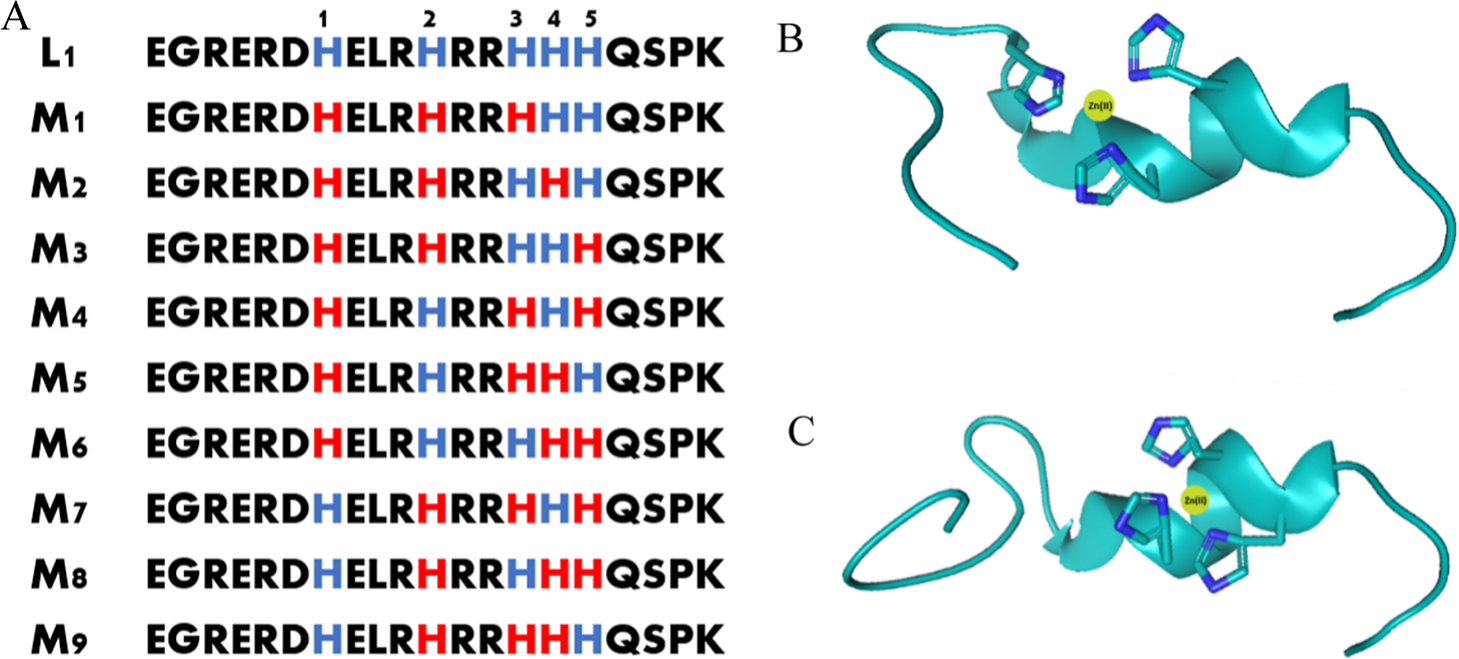
Proposed binding modes for the zinc(II) complexes with L1 (EGRERDHELRHRRHHHQSPK) at pH 7.40: (A) all of the possible models (M_1_–M_9_), where three His residues binding the Zn(II) ion are marked in red; (B) schematic model for M_1_, where imidazoles from His1, His2, and His3 can bind the Zn(II) ion; (C) schematic model for M_9_, where imidazoles from His2, His3, and His4 can bind the Zn(II) ion. Figures B and C were generated using PyMOL. The yellow sphere represents the Zn(II) ion.

The first complex species that appears at acidic pH, [ZnH_5_L]^2+^, reaches its maximum concentration at pH 5.70 (Figure 8A). Most likely, at this pH, Zn(II) ions are already bound by imidazole nitrogens from three His residues ({3N_im_} donor set). For the next complex species: [ZnH_4_L]^+^ and [ZnH_3_L], lower p*K*_a_ values were observed in relation to those in the ligand, and the occurrence of polymorphic binding sites with different sets of three His residues {3N_im_} is suggested (Figure 9). The coordination mode remains unchanged up to the [ZnH_2_L]^−^ complex species, which reaches its maximum concentration at pH 7.40. Here, the p*K*_a_ value 7.89 may be associated with the coordination of the N-terminal amine group resulting in the {3N_im_, 1NH_2_} donor set. The next two complex species [ZnHL]^2–^ and [ZnL]^3–^ with p*K*_a_ 8.87 and p*K*_a_ 9.68, respectively, can be attributed to the deprotonation of two aqua ligands bound to the central Zn(II) ion. The appearance of the last complex species, [ZnH_–2_L]^5–^, can be associated with the deprotonation of nonbonding Lys and Arg residues.

#### Zn(II)–L2 (EGRERDHELRHRR) System

The formation of the first complex species [ZnHL]^−^ (with the maximum concentration at pH 6.50, Figure 8B) can be attributed to the deprotonation and coordination of two imidazole nitrogen atoms. The next complex species, [ZnL]^2–^, is associated with the coordination of nitrogen atom from the N-terminal amine group, resulting in a {2N_im_, 1NH_2_} donor set, which is supported by the significant decrease in the corresponding p*K*_a_ values: p*K*_a_ = 6.88 (in the complex) to p*K*_a_ = 7.56 (in the ligand). The appearance of the last complex species, [ZnH_–1_L]^3–^ (with the maximum concentration at pH 8.61), is most likely related to the deprotonation of a coordinated aqua ligand.

#### Zn(II)–L3 (HHHQSPK) System

In the first complex species detected at acidic pH, [ZnHL]^2+^ with a maximum concentration at pH 6.40 (Figure 8C), the coordination of Zn(II) to a maximum of four nitrogen atoms: (i) three from histidyl residues and (ii) one from the N-terminus of the peptide, is suggested. However, the possibility of such a coordination mode is low for steric reasons (three adjacent His residues). The existence of at least two species in equilibrium (polymorphic states) is quite probable here, involving different donor sets with a maximum of two His residues and an amine nitrogen {2N_im_, 1NH_2_}. The next species, [ZnL]^+^, is probably formed as a result of the deprotonation of an aqua ligand, coordinated to the central Zn(II) ion, resulting in the {2N_im_, 1NH_2_, 1OH^–^} set of donor atoms. The formation of the next complex species, [ZnH_–1_L]^−^, is probably connected with the deprotonation of a second aqua ligand coordinated to the Zn(II) ion, leading to the formation of a less common structure, such as a square pyramid, in which the zinc ion is coordinated to five donors {2N_im_, 1NH_2_, 2OH^–^}.^59^ The last species, [ZnH_–2_L]^2–^, is formed by deprotonation of the Lys residue (p*K*_a_ = 10.33 → p*K*_a_ = 10.49) and does not affect the complex binding mode.

**Figure 10:**
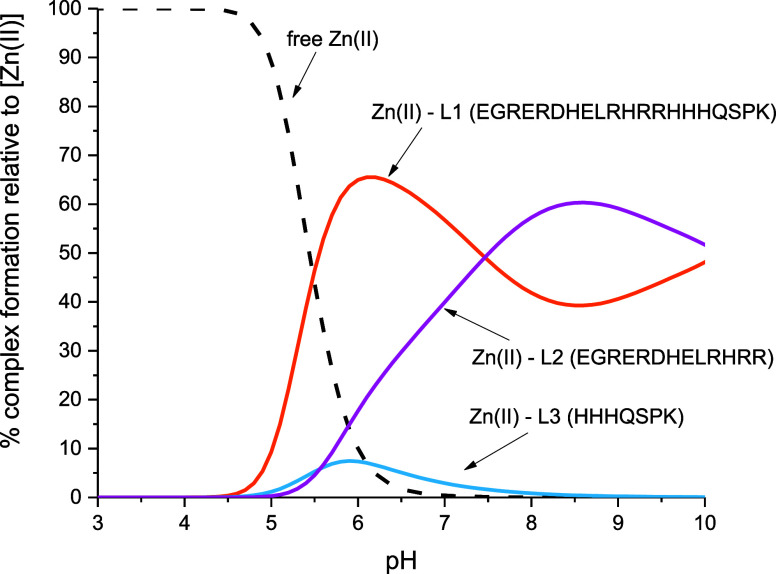
Competition plot for Zn(II) complexes with MUC7 fragments: L1—EGRERDHELLRHRRHHHQSPK (orange), L2—EGRERDHELLRHRR (pink), and L3—HHHQSPK (blue) based on potentiometric data (Table 1), describing complex formation at different pH values in a hypothetical situation, in which equimolar amounts of all the reagents are mixed. Conditions: *T* = 298 K, [Cu(II)] = [L1] = [L2] = [L3] = 0.001 M.

The competition plot for L1 (EGRERDHELRHRRHHHQSPK), L2 (EGRERDHELRHRR), and L3 (HHHQSPK) ligands with zinc(II) ions (Figure 10) shows that Zn(II) binds more effectively to L1 than to L2 in the pH range of 4.50–7.50, most likely due to the presence of polymorphic binding sites in the case of the Zn(II)–L1 system. However, above pH 7.50, a slightly higher efficiency of Zn(II) binding to L2 is observed (though the difference is minor and it can be said that the complexes have a comparable stability). Therefore, it can be concluded that L1 and L2 have a higher affinity in Zn(II) ion binding than L3, which is opposite to Cu(II) complexes in this pH range (Figure 7A). Therefore, it can be suggested that the N-terminal HHH region is a tempting site for Cu(II) but not for Zn(II) ions.

### Structural Characterization of MUC7 Fragments and Their Complexes by Far-UV CD Experiments

**Figure 11:**
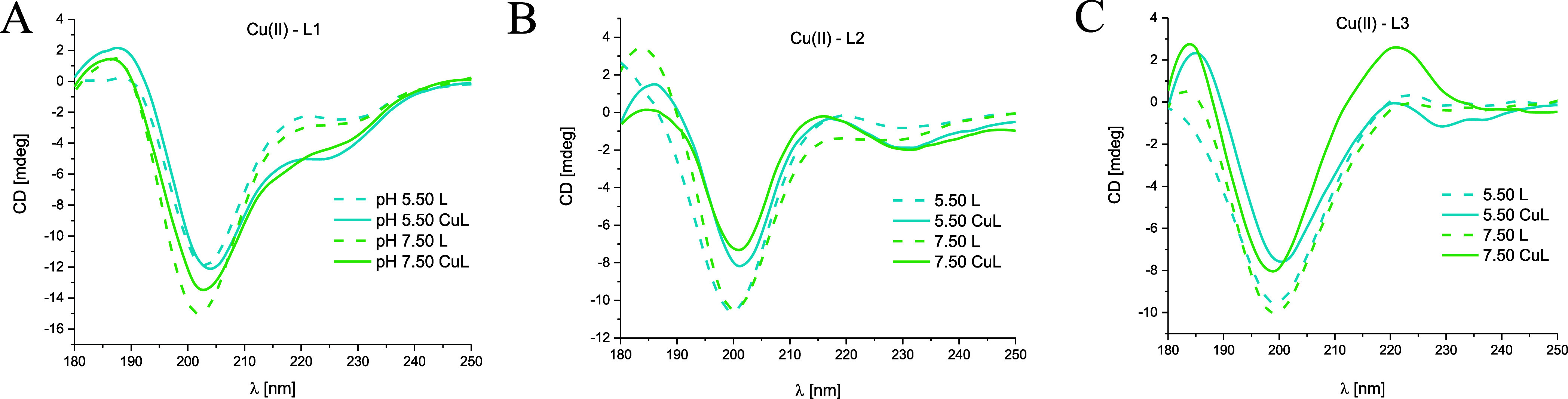
Far-UV CD spectra at 180–250 nm at pH 5.50 and 7.50 for (A) Cu(II)–L1 (EGRERDHELRHRRHHHQSPK), (B) Cu(II)–L2 (EGRERDHELRHRR), and (C) Cu(II)–L3 (HHHQSPK) systems in an aqueous solution of 4 mM HClO_4_, with *I* = 0.1 M NaClO_4_; molar ratio M/L 0.8:1; the optical path length = 0.2 mm; *C*_L_ = 0.3 mM. The dashed lines correspond to the peptide spectra.

**Figure 12:**
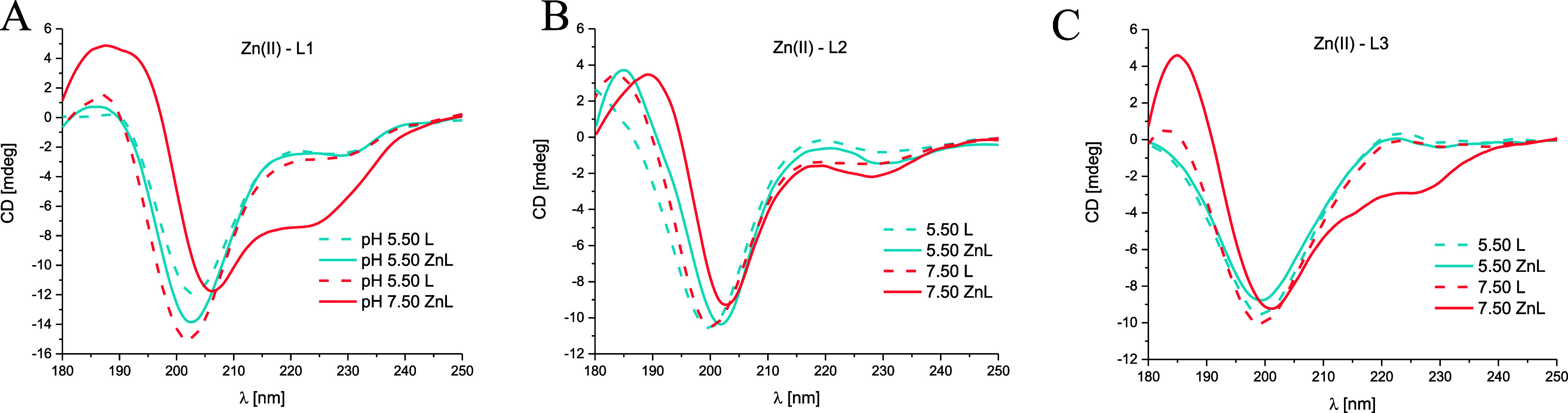
Far-UV CD spectra at 180–250 nm at pH 5.50 and 7.50 for (A) Zn(II)–L1 (EGRERDHELRHRRHHHQSPK), (B) Zn(II)–L2 (EGRERDHELRHRR), and (C) Zn(II)–L3 (HHHQSPK) systems in an aqueous solution of 4 mM HClO_4_, with *I* = 0.1 M NaClO_4_; molar ratio M/L 0.8:1; the optical path length = 0.2 mm; *C*_L_ = 0.3 mM. The dashed lines correspond to the peptide spectra.

Far-UV CD spectra for MUC7 fragments and their complexes were recorded in the wavelength range λ = 180–250 nm for the chosen pH values in an aqueous solution. The obtained results are presented in Figures 11 and 12.

The obtained far-UV CD spectra show the occurrence of a random-coil conformation in the case of the Cu(II)–EGRERDHELRHRR (L2) and Cu(II)–HHHQSPK (L3) systems (Figure 11B,C). Therefore, it can be concluded that the coordination of Cu(II) ions does not influence the change of the secondary structure of these peptides. However, in the case of Cu(II)–EGRERDHELRHRRHHQSPK (L1), the tendency to form a helical structure was observed at pH 7.50, indicating that copper ions influence the secondary structure of L1 peptide.

The obtained spectra at pH 5.50 for the MUC7 fragments and their Zn(II) complexes do not suggest the existence of any ordered secondary structures (Figure 12). However, around pH 7.50, the tendency to induce a helical structure was observed in the case of Zn(II)–L1 and Zn(II)–L3 systems. A more pronounced effect of Zn(II) ions on the helical structure formation, with typical bands at 223, 206, and 188 mm, was observed in the case of the L1 ligand.

### Antimicrobial Activity

To assess the antimicrobial effectiveness of the MUC7 fragments and their metal ion complexes, a broth microdilution test was employed. This technique enabled the identification of the MIC, which represents the minimum inhibitory concentration at which the growth of the tested microorganisms is restrained. Keeping in mind that the pH of saliva is typically slightly acidic, ranging from 5.00 to 8.00^60^ and varying among individuals’ health conditions, we evaluated the antimicrobial activity of our MUC7 fragments and their metal complexes on six bacterial strains and one fungal strain at pH 5.40 and 7.40 (Tables S3 and S4).

MUC7 peptides and their complexes show antimicrobial activity only against *S. sanguinis* (Tables 2, S3, and S4), which makes biological sense, since this bacterium occurs in the oral cavity under physiological conditions and, but during certain opportunities, such as invasive dental procedures, *S. sanguinis* can potentially enter the bloodstream and establish colonies on the heart valves, which may become a primary contributor to infective endocarditis.^61,62^

**Table 2:** *In Vitro* Antibacterial Activity of Peptides and Complexes Determined as a Minimal Inhibitory Concentration (MIC) (μg/mL); Antimicrobial Assays Were Performed in 10 mM MES Buffer (pH 5.40) and 10 mM HEPES Buffer (pH 7.40)^a^

	*S. sanguinis* PMC 2335 pH 5.40	*S. sanguinis* PMC 2335 pH 7.40
L1	n/d	n/d
Cu(II)–L1	n/d	n/d
Zn(II)–L1	n/d	**500**
L2	n/d	n/d
Cu(II)–L2	**250**	n/d
Zn(II)–L2	**250**	**500**
L3	**250**	**500**
Cu(II)–L3	**250**	n/d
Zn(II)–L3	**125**	**250**

a Experiments were performed for all peptides and their copper(II) and zinc(II) complexes. n/d, not determined.

The antimicrobial activity of mucin fragments is evidently influenced by both pH levels and the presence of Zn(II). At pH 7.40 (Table 2), L1 (EGRERDHELRHRRHHHQSPK) and L2 (EGRERDHELRHRR) show antimicrobial activity only after binding to Zn(II) ions. Native L3 (HHHQSPK) itself has antibacterial activity, but after binding zinc ions, an increase of antimicrobial activity is observed (MIC 500 μg/mL → 250 μg/mL). The metal ions, copper and zinc, have shown no antibacterial activity against Gram-positive bacteria, Gram-negative bacteria, and *C. albicans* strains at the concentration range of 0.3 to 38 μg/mL (Tables S3 and S4).

The general Zn(II)-induced increase of antimicrobial activity of the studied MUC7 fragments is probably related to the local change of the complex charge and, in addition, the structural change observed at pH 7.40 for peptides L1 and L2 after Zn(II) ion binding (Figure 12).

Again, as in one of our previous works^33^ we point out that the strong local positive charge in the metal-bound polyhistidine motif correlates well with its antimicrobial activity—a strongly positively charged complex will have a strong affinity for the negatively charged cell membrane of the pathogen.

## Conclusions

Metal–AMP complexes are chemically fascinating and biologically underestimated phenomenon. Some show great potential as candidates for antimicrobial treatments, while others are an elegant explanation of why nature has decided to keep a relatively high level of a specific metal ion in a given biological compartment.

First, it is worth mentioning that the antimicrobial activity of the studied mucins and their metal complexes strongly depends on pH—they are more effective against *S. sanguinis*, a common oral cavity pathogen at pH 5.40 than at pH 7.40—this makes biological sense, keeping in mind that the saliva pH is often acidic. Second, the antimicrobial potency of natural hydrolysis fragments of MUC7 against *S. sanguinis* is either enhanced or triggered by the coordination of Zn(II). The MIC values (usually 125–250 μg/mL) do not hold any real promise of a pharmaceutically applicable potential; however, Zn(II) coordination seems to be nature’s choice to modulate the antimicrobial activity of MUC7 fragments by changing their local charge (as observed for semenogelin complexes^33^), and, in the case of L1 (EGRERDHELRHRRHHHQSPK) and L3 (HHHQSPK)—by inducing a structural change that contributes to antimicrobial potency enhancement. Additionally, the binding of the metal may favor interaction with the bacterial cell wall and the components of the membrane, cytoplasmic leakage, detachment of cytoplasm from the cell membrane, and reduced density of the lipid bilayer; however, additional experiments need to be done to confirm these findings.

## Supporting Information Available

Mass spectra for all metal–peptide systems; EPR spectra for Cu(II)–L1 and Cu(II)–L2 systems; thermodynamic and spectroscopic data for proton and the Cu(II)–MUC7 fragments; thermodynamic data for proton and the Zn(II)–MUC7 fragments; and *in vitro* antibacterial activity of peptides/complexes determined as a minimal inhibitory concentration (MIC) (μg/mL) at pH 5.40 and 7.40 (PDF)

## References

[ref1] Kim Y.; Kim Y.; Kho H. Effects of Smoking on Trace Metal Levels in Saliva. Oral Dis. 2010, 16 (8), 823–830. 10.1111/j.1601-0825.2010.01698.x. 20604873

[ref2] Humphrey S. P.; Williamson R. T. A Review of Saliva: Normal Composition, Flow, and Function. J. Prosthet. Dent. 2001, 85 (2), 162–169. 10.1067/mpr.2001.113778. 11208206

[ref3] Khurshid Z.; Naseem M.; Sheikh Z.; Najeeb S.; Shahab S.; Zafar M. S. Oral Antimicrobial Peptides: Types and Role in the Oral Cavity. Saudi Pharmaceut. J. 2016, 24 (5), 515–524. 10.1016/j.jsps.2015.02.015. PMC5059823 27752223

[ref4] Frenkel E. S.; Ribbeck K. Salivary Mucins in Host Defense and Disease Prevention. J. Oral Microbiol. 2015, 7 (1), 29759 10.3402/jom.v7.29759. 26701274 PMC4689954

[ref5] Tabak L. A. In Defense of the Oral Cavity: Structure, Biosynthesis, and Function of Salivary Mucins. Annu. Rev. Physiol. 1995, 57 (1), 547–564. 10.1146/annurev.ph.57.030195.002555. 7778877

[ref6] Brown R. B.; Hollingsworth M. A. Mucin Family of Glycoproteins. In Encyclopedia of Biological Chemistry; Elsevier, 2013; pp 200–204.

[ref7] Liu B.; Rayment S.; Oppenheim F. G.; Troxler R. F. Isolation of Human Salivary Mucin MG2 by a Novel Method and Characterization of Its Interactions with Oral Bacteria. Arch. Biochem. Biophys. 1999, 364 (2), 286–293. 10.1006/abbi.1999.1141. 10190986

[ref8] Janicka-Kłos A.; Czapor-Irzabek H.; Janek T. The Potential Antimicrobial Action of Human Mucin 7 15-Mer Peptide and Its Metal Complexes. Int. J. Mol. Sci. 2022, 23 (1), 418 10.3390/ijms23010418. PMC8745124 35008844

[ref9] Bobek L. A.; Situ H. MUC7 20-Mer: Investigation of Antimicrobial Activity, Secondary Structure, and Possible Mechanism of Antifungal Action. Antimicrob. Agents Chemother. 2003, 47 (2), 643–652. 10.1128/AAC.47.2.643-652.2003. 12543672 PMC151741

[ref10] Wei G.-X.; Bobek L. A. Human Salivary Mucin MUC7 12-Mer-land 12-Mer-dPeptides: Antifungal Activity in Saliva, Enhancement of Activity with Protease Inhibitor Cocktail or EDTA, and Cytotoxicity to Human Cells. Antimicrob. Agents Chemother. 2005, 49 (6), 2336–2342. 10.1128/AAC.49.6.2336-2342.2005. 15917530 PMC1140489

[ref11] Liu B.; Rayment S. A.; Gyurko C.; Oppenheim F. G.; Offner G. D.; Troxler R. F. The Recombinant N-Terminal Region of Human Salivary Mucin MG2 (MUC7) Contains a Binding Domain for Oral Streptococci and Exhibits Candidacidal Activity. Biochem. J. 2000, 345 (3), 557–564. 10.1042/bj3450557. 10642514 PMC1220790

[ref12] Gusman H.; Lendenmann U.; Grogan J.; Troxler R. F.; Oppenheim F. G. Is Salivary Histatin 5 a Metallopeptide?. Biochim. Biophys. Acta, Protein Struct. Mol. Enzymol. 2001, 1545 (1–2), 86–95. 10.1016/S0167-4838(00)00265-X. 11342034

[ref13] Łoboda D.; Kozłowski H.; Rowińska-Żyrek M. Antimicrobial Peptide-Metal Ion Interactions-a Potential Way of Activity Enhancement. New J. Chem. 2018, 42, 7560–7568. 10.1039/c7nj04709f.

[ref14] Walkenhorst W. F.; Sundrud J. N.; Laviolette J. M. Additivity and Synergy between an Antimicrobial Peptide and Inhibitory Ions. Biochim. Biophys. Acta, Biomembr. 2014, 1838 (9), 2234–2242. 10.1016/j.bbamem.2014.05.005. 24841756

[ref15] Dudek D.; Garstka K.; Miller A.; Dzień E.; Wątły J.; Hecel A.; Rowińska-Żyrek M. The Interaction of Antimicrobial Peptides With Metal Ions - the Relationship Between Coordination Chemistry, Structure, Thermodynamics and Mode of Action. Wiadomości Chemiczne 2022, 76, 365–391. 10.53584/wiadchem.2022.5.7.

[ref16] Hood M. I.; Skaar E. P. Nutritional Immunity: Transition Metals at the Pathogen-Host Interface. Nat. Rev. Microbiol. 2012, 10 (8), 525–537. 10.1038/nrmicro2836. 22796883 PMC3875331

[ref17] Miller A.; Matera-Witkiewicz A.; Mikołajczyk A.; Wieczorek R.; Rowińska-Żyrek M. Chemical “Butterfly Effect” Explaining the Coordination Chemistry and Antimicrobial Properties of Clavanin Complexes. Inorg. Chem. 2021, 60 (17), 12730–12734. 10.1021/acs.inorgchem.1c02101. 34382773 PMC8424629

[ref18] Dudek D.; Dzień E.; Wątły J.; Matera-Witkiewicz A.; Mikołajczyk A.; Hajda A.; Olesiak-Bańska J.; Rowińska-Żyrek M. Zn(II) Binding to Pramlintide Results in a Structural Kink, Fibril Formation and Antifungal Activity. Sci. Rep. 2022, 12 (1), 20543 10.1038/s41598-022-24968-y. 36446825 PMC9708664

[ref19] Wątły J.; Szarszoń K.; Mikołajczyk A.; Grelich-Mucha M.; Matera-Witkiewicz A.; Olesiak-Bańska J.; Rowińska-Żyrek M. Zn(II) Induces Fibril Formation and Antifungal Activity in Shepherin I, An Antimicrobial Peptide from *Capsella Bursa-Pastoris*. Inorg. Chem. 2023, 62, 19786–19794. 10.1021/acs.inorgchem.3c03409. 37983127 PMC10698721

[ref20] Situ H.; Bobek L. A. In Vitro Assessment of Antifungal Therapeutic Potential of Salivary Histatin-5, Two Variants of Histatin-5, and Salivary Mucin (MUC7) Domain 1. Antimicrob. Agents Chemother. 2000, 44 (6), 1485–1493. 10.1128/AAC.44.6.1485-1493.2000. 10817697 PMC89901

[ref21] Mehrotra R.; Thornton D. J.; Sheehan J. K. Isolation and Physical Characterization of the MUC7 (MG2) Mucin from Saliva: Evidence for Self-Association. Biochem. J. 1998, 334 (2), 415–422. 10.1042/bj3340415. 9716500 PMC1219704

[ref22] Kozłowski H.; Kowalik-Jankowska T.; Jeżowska-Bojczuk M. Chemical and Biological Aspects of Cu2+ Interactions with Peptides and Aminoglycosides. Coord. Chem. Rev. 2005, 249 (21–22), 2323–2334. 10.1016/j.ccr.2005.04.027.

[ref23] Zoroddu M. A.; Medici S.; Peana M.; Anedda R. NMR Studies of Zinc Binding in a Multi-Histidinic Peptide Fragment. Dalton Trans. 2010, 39 (5), 1282–1294. 10.1039/B914296G. 20104355

[ref24] Sóvágó I.; Ősz K. Metal Ion Selectivity of Oligopeptides. Dalton Trans. 2006, (32), 3841–3854. 10.1039/B607515K. 16896443

[ref25] Harford C.; Sarkar B. Amino Terminal Cu(II)- and Ni(II)-Binding (ATCUN) Motif of Proteins and Peptides: Metal Binding, DNA Cleavage, and Other Properties. Acc. Chem. Res. 1997, 30 (3), 123–130. 10.1021/ar9501535.

[ref26] Migliorini C.; Witkowska D.; Valensin D.; Kamysz W.; Kozlowski H. Competition between Histamine-like and Poly-Imidazole Coordination Sites for Cu2+ and Zn2+ Ions in Zebra-Fish Peptide of Prion-like Protein. Dalton Trans. 2010, 39 (37), 8663 10.1039/c0dt00137f. 20714613

[ref27] Libardo M. D.; Cervantes J. L.; Salazar J. C.; Angeles-Boza A. M. Improved Bioactivity of Antimicrobial Peptides by Addition of Amino-Terminal Copper and Nickel (ATCUN) Binding Motifs. ChemMedChem 2014, 9, 1892–1901. 10.1002/cmdc.201402033. 24803240 PMC4440792

[ref28] Miyamoto T.; Kamino S.; Odani A.; Hiromura M.; Enomoto S. Basicity of N-Terminal Amine in ATCUN Peptide Regulates Stability Constant of Albumin-like Cu ^2+^ Complex. Chem. Lett. 2013, 42 (9), 1099–1101. 10.1246/cl.130405.

[ref29] Miyamoto T.; Fukino Y.; Kamino S.; Ueda M.; Enomoto S. Enhanced Stability of Cu ^2+^ -ATCUN Complexes under Physiologically Relevant Conditions by Insertion of Structurally Bulky and Hydrophobic Amino Acid Residues into the ATCUN Motif. Dalton Trans. 2016, 45 (23), 9436–9445. 10.1039/C6DT01387B. 27184978

[ref30] Maiti B. K.; Govil N.; Kundu T.; Moura J. J. G. Designed Metal-ATCUN Derivatives: Redox- and Non-Redox-Based Applications Relevant for Chemistry, Biology, and Medicine. iScience 2020, 23 (12), 101792 10.1016/j.isci.2020.101792. 33294799 PMC7701195

[ref31] Livera C. E.; Pettit L. D.; Bataille M.; Perly B.; Kozlowski H.; Radomska B. A Thermodynamic and Spectroscopic Study of the Proton and Copper(II) Complexes of L-Prolyl-L-Histidine, D-Prolyl-L-Histidine, L-Histidyl-L-Histidine, and D-Histidyl-L-Histidine. J. Chem. Soc., Dalton Trans. 1987, (3), 661 10.1039/dt9870000661.

[ref32] Kozłowski H.; Bal W.; Dyba M.; Kowalik-Jankowska T. Specific Structure-Stability Relations in Metallopeptides. Coord. Chem. Rev. 1999, 184 (1), 319–346. 10.1016/S0010-8545(98)00261-6.

[ref33] Dudek D.; Miller A.; Hecel A.; Kola A.; Valensin D.; Mikołajczyk A.; Barcelo-Oliver M.; Matera-Witkiewicz A.; Rowińska-Żyrek M. Semenogelins Armed in Zn(II) and Cu(II): May Bioinorganic Chemistry Help Nature to Cope with *Enterococcus Faecalis* ?. Inorg. Chem. 2023, 62, 14103–14115. 10.1021/acs.inorgchem.3c02390. 37582221 PMC10466376

[ref34] Menach E.; Hashida Y.; Yasukawa K.; Inouye K. Effects of Conversion of the Zinc-Binding Motif Sequence of Thermolysin, HEXXH, to That of Dipeptidyl Peptidase III, HEXXXH, on the Activity and Stability of Thermolysin. Biosci. Biotechnol. Biochem. 2013, 77 (9), 1901–1906. 10.1271/bbb.130360. 24018667

[ref35] Gran G.; Dahlenborg H.; Laurell S.; Rottenberg M. Determination of the Equivalent Point in Potentiometric Titrations. Acta Chem. Scand. 1950, 4, 559–577. 10.3891/acta.chem.scand.04-0559.

[ref36] Gans P.; Sabatini A.; Vacca A. Investigation of Equilibria in Solution. Determination of Equilibrium Constants with the HYPERQUAD Suite of Programs. Talanta 1996, 43 (10), 1739–1753. 10.1016/0039-9140(96)01958-3. 18966661

[ref37] Petitt L. IUPAC Stability Constants Database. Chem. Int. 2001, 23 (1), 18 10.1515/ci.2001.23.1.18b.

[ref38] Arena G.; Cali R.; Rizzarelli E.; Sammartano S. Thermodynamic Study on the Formation of the Cupric Ion Hydrolytic Species. Thermochim. Acta 1976, 16 (3), 315–321. 10.1016/0040-6031(76)80024-X.

[ref39] Alderighi L.; Gans P.; Ienco A.; Peters D.; Sabatini A.; Vacca A. Hyperquad Simulation and Speciation (HySS): A Utility Program for the Investigation of Equilibria Involving Soluble and Partially Soluble Species. Coord. Chem. Rev. 1999, 184 (1), 311–318. 10.1016/S0010-8545(98)00260-4.

[ref40] Gillum A. M.; Tsay E. Y. H.; Kirsch D. R. Isolation of the Candida Albicans Gene for Orotidine-5′-Phosphate Decarboxylase by Complementation of S. Cerevisiae Ura3 and E. Coli PyrF Mutations. Mol. Gen. Genet. 1984, 198 (1), 179–182. 10.1007/BF00328721. 6394964

[ref41] Wiegand I.; Hilpert K.; Hancock R. E. W. Agar and Broth Dilution Methods to Determine the Minimal Inhibitory Concentration (MIC) of Antimicrobial Substances. Nat. Protoc. 2008, 3 (2), 163–175. 10.1038/nprot.2007.521. 18274517

[ref42] Perinelli M.; Guerrini R.; Albanese V.; Marchetti N.; Bellotti D.; Gentili S.; Tegoni M.; Remelli M. Cu(II) Coordination to His-Containing Linear Peptides and Related Branched Ones: Equalities and Diversities. J. Inorg. Biochem. 2020, 205, 110980 10.1016/j.jinorgbio.2019.110980. 31931375

[ref43] Lakatos A.; Gyurcsik B.; Nagy N. V.; Csendes Z.; Wéber E.; Fülöp L.; Kiss T. Histidine-rich branched peptides as Cu(ii) and Zn(ii) chelators with potential therapeutic application in Alzheimer’s disease. Dalton Trans. 2012, 41 (6), 1713–1726. 10.1039/C1DT10989H. 22159144

[ref44] Watly J.; Simonovsky E.; Barbosa N.; Spodzieja M.; Wieczorek R.; Rodziewicz-Motowidlo S.; Miller Y.; Kozlowski H. African Viper Poly-His Tag Peptide Fragment Efficiently Binds Metal Ions and Is Folded into an α-Helical Structure. Inorg. Chem. 2015, 54 (16), 7692–7702. 10.1021/acs.inorgchem.5b01029. 26214303

[ref45] Fitch C. A.; Platzer G.; Okon M.; Garcia-Moreno E B.; McIntosh L. P. Arginine: Its p *K*_a_ Value Revisited. Protein Sci. 2015, 24 (5), 752–761. 10.1002/pro.2647. 25808204 PMC4420524

[ref46] Grimsley G. R.; Scholtz J. M.; Pace C. N. A Summary of the Measured p *K* Values of the Ionizable Groups in Folded Proteins. Protein Sci. 2009, 18 (1), 247–251. 10.1002/pro.19. 19177368 PMC2708032

[ref47] Pettit L. D.; Gregor J. E.; Kozłowski H. Complex Formation between Metal Ions and Peptides. In Perspectives on Bioinorganic Chemistry; Hay R. W., Dilworth J. R., Nolan K. B., Eds.; JAI Press: London, 1991; Vol. 1, pp 1–41.

[ref48] Kowalik-Jankowska T.; Ruta-Dolejsz M.; Wiśniewska K.; Łankiewicz L.; Kozłowski H. Copper(II) complexation by human and mouse fragments (11–16) of β-amyloid peptide. J. Chem. Soc., Dalton Trans. 2000, (24), 4511–4519. 10.1039/b006125p.

[ref49] Witkowska D.; Politano R.; Rowinska-Zyrek M.; Guerrini R.; Remelli M.; Kozlowski H. The Coordination of Ni ^II^ and Cu ^II^ Ions to the Polyhistidyl Motif of Hpn Protein: Is It as Strong as We Think?. Chem.—Eur. J. 2012, 18 (35), 11088–11099. 10.1002/chem.201200780. 22829429

[ref50] Bellotti D.; Tocchio C.; Guerrini R.; Rowińska-Żyrek M.; Remelli M. Thermodynamic and Spectroscopic Study of Cu(ii) and Zn(ii) Complexes with the (148–156) Peptide Fragment of C4YJH2, a Putative Metal Transporter of *Candida Albicans*. Metallomics 2019, 11 (12), 1988–1998. 10.1039/C9MT00251K. 31737884

[ref51] Farkas E.; Sóvágó I.; Kiss T.; Gergely A. Studies on transition-metal–peptide complexes. Part 9. Copper(II) complexes of tripeptides containing histidine. J. Chem. Soc., Dalton Trans. 1984, (4), 611–614. 10.1039/DT9840000611.

[ref52] Eury H.; Bijani C.; Faller P.; Hureau C. Copper(II) Coordination to Amyloid β: Murine versus Human Peptide. Angew. Chem., Int. Ed. 2011, 50 (4), 901–905. 10.1002/anie.201005838. 21246687

[ref53] Guilloreau L.; Damian L.; Coppel Y.; Mazarguil H.; Winterhalter M.; Faller P. Structural and thermodynamical properties of CuII amyloid-β16/28 complexes associated with Alzheimer’s disease. J. Biol. Inorg. Chem. 2006, 11 (8), 1024–1038. 10.1007/s00775-006-0154-1. 16924555

[ref54] Mylonas M.; Krężel A.; Plakatouras J. C.; Hadjiliadis N.; Bal W. Interactions of Transition Metal Ions with His-Containing Peptide Models of Histone H2A. J. Mol. Liq. 2005, 118 (1–3), 119–129. 10.1016/j.molliq.2004.07.025. PMC2267071 18365073

[ref55] Mylonas M.; Plakatouras J. C.; Hadjiliadis N.; Krężel A.; Bal W. Potentiometric and Spectroscopic Studies of the Interaction of Cu(II) Ions with the Hexapeptides AcThrAlaSerHisHisLysNH2, AcThrGluAlaHisHisLysNH2, AcThrGluSerAlaHisLysNH2 and AcThrGluSerHisAlaLysNH2, Models of C-Terminal Tail of Histone H2A. Inorg. Chim. Acta 2002, 339, 60–70. 10.1016/S0020-1693(02)00925-8.

[ref56] Sokolowska M.; Krezel A.; Dyba M.; Szewczuk Z.; Bal W. Short Peptides Are Not Reliable Models of Thermodynamic and Kinetic Properties of the N-Terminal Metal Binding Site in Serum Albumin. Eur. J. Biochem. 2002, 269 (4), 1323–1331. 10.1046/j.1432-1033.2002.02772.x. 11856367

[ref57] Wa̧tły J.; Hecel A.; Wieczorek R.; Rowińska-Żyrek M.; Kozłowski H. Poly-Gly Region Regulates the Accessibility of Metal Binding Sites in Snake Venom Peptides. Inorg. Chem. 2022, 61 (36), 14247–14251. 10.1021/acs.inorgchem.2c02584. 36039984 PMC9472272

[ref58] Balogh B. D.; Bihari Z.; Buglyó P.; Csire G.; Kerekes Z.; Lukács M.; Sóvágó I.; Várnagy K. Metal Binding Selectivity of an N-Terminally Free Multihistidine Peptide HAVAHHH-NH _2_. New J. Chem. 2019, 43 (2), 907–916. 10.1039/C8NJ04538K.

[ref59] Wątły J.; Hecel A.; Rowińska-Żyrek M.; Kozłowski H. Impact of Histidine Spacing on Modified Polyhistidine Tag - Metal Ion Interactions. Inorg. Chim. Acta 2018, 472, 119–126. 10.1016/j.ica.2017.06.053.

[ref60] Galgut P. N. The Relevance of PH to Gingivitis and Periodontitis. J. Int. Acad. Periodontol. 2001, 3 (3), 61–67. 12666943

[ref61] Sumioka R.; Nakata M.; Okahashi N.; Li Y.; Wada S.; Yamaguchi M.; Sumitomo T.; Hayashi M.; Kawabata S. Streptococcus Sanguinis Induces Neutrophil Cell Death by Production of Hydrogen Peroxide. PLoS One 2017, 12 (2), e0172223 10.1371/journal.pone.0172223. 28222125 PMC5319702

[ref62] Moreillon P.; Que Y.-A. Infective Endocarditis. Lancet 2004, 363 (9403), 139–149. 10.1016/S0140-6736(03)15266-X. 14726169

