# Supplementary material for: Insights into the Chemistry, Structure, and Biological Activity of Human Salivary MUC7 Fragments and Their Cu(II) and Zn(II) Complexes
Source: Inorg Chem. 2024 Jun 10;63(25):11616–27. doi: 10.1021/acs.inorgchem.4c00868 (PMC11200262; doi:10.1021/acs.inorgchem.4c00868)
Supplement: Supplementary file 1 — ic4c00868_si_001.pdf [file ic4c00868_si_001.pdf]

Supporting Information for:

Insight into the chemistry, structure and the biological activity of human salivary MUC7 fragments and their Cu(II) and Zn(II) complexes

*Klaudia Szarszoń[†], Silke Andrä[†], Tomasz Janek[‡], Joanna Wątły[†]**

[†]Faculty of Chemistry, University of Wrocław, F. Joliot-Curie 14, 50-383 Wrocław, Poland

[‡]Department of Biotechnology and Food Microbiology, Wrocław University of Environmental and Life Sciences, Chełmońskiego 37, 51-630, Wrocław, Poland

TABLE OF CONTENTS:

Figure S1. Mass spectra (ESI-MS) for the system Cu(II) – L1 (EGRERDHELRHRRHHHQSPK) A) whole spectrum; B) example of experimental and simulated spectrum for $[CuL]^{4+}$ complex with 1:1 stoichiometry. 3

Figure S2. Mass spectra (ESI-MS) for the system Zn(II) – L1 (EGRERDHELRHRRHHHQSPK) A) whole spectrum; B) example of experimental and simulated spectrum for $[ZnL]^{3+}$ complex with 1:1 stoichiometry. 4

Figure S3. Mass spectra (ESI-MS) for the system Cu(II) – L2 (EGRERDHELRHRR) A) whole spectrum; B) example of experimental and simulated spectrum for $[CuL+Na+K]^{3+}$ complex; C) example of experimental and simulated spectrum for $[CuL]^{3+}$ complex with 1:1 stoichiometry. 5

Figure S4. Mass spectra (ESI-MS) for the system Zn(II) – L2 (EGRERDHELRHRR) A) whole spectrum; B) example of experimental and simulated spectrum for $[ZnL]^{3+}$ complex with 1:1 stoichiometry. 6

Figure S5. Mass spectra (ESI-MS) for the system Cu(II) – HHHQSPK A) whole spectrum; B) example of experimental and simulated spectrum for $[CuL+K]^{2+}$ complex; C) example of experimental and simulated spectrum for $[CuL+Na]^{2+}$ complex with 1:1 stoichiometry. 7

Figure S6. Mass spectra (ESI-MS) for the system Zn(II) – HHHQSPK A) whole spectrum; B) example of experimental and simulated spectrum for $[ZnL+K]^{2+}$ complex with 1:1 stoichiometry...............................8

Figure S7. pH-dependent EPR spectra for the Cu(II) - L1 (EGRERDHELRHRRHHHQSPK) system in aqueous solution with the addition of ethylene glycol (30%) at temperature 77 K. [Cu(II)] = 0.001 M, molar ratio M:L equal to 0.8:1. 9

Figure S8. pH-dependent EPR spectra for the Cu(II) – L2 (EGRERDHELRHRR) system in aqueous solution with the addition of ethylene glycol (30%) at temperature 77 K. Cu(II) concentration = 0.001 M, molar ratio M:L equal to 0.8:1. 9

Table S1. Thermodynamic and spectroscopic data for proton and the Cu(II) – MUC7 fragments in aqueous solution of 4 mM $HClO_4$ for each calculated complex species with the proposed coordination modes. C_L = 0.4 mM; molar ratio M:L – 0.8:1; I = 0.1 M $NaClO_4$; T = 25 C. 9

Table S2. Thermodynamic data for proton and the Zn(II) – MUC7 fragments in aqueous solution of 4 mM $HClO_4$ for each calculated complex species with the proposed coordination modes. C_L = 0.4 mM; molar ratio M:L – 0.8:1; I = 0.1 M $NaClO_4$; T = 25 C. 12

Table S3. *In vitro* antibacterial activity of peptides/complexes determined as a minimal inhibitory concentration (MIC) (μg/mL); antimicrobial assays were performed in 10 mM MES buffer (pH 5.40). Experiments were performed for all peptides and their copper(II) and zinc(II) complexes. ND, not determined. 13
Table S4. *In vitro* antibacterial activity of peptides/complexes determined as a minimal inhibitory concentration (MIC) (μg/mL); antimicrobial assays were performed in 10 mM HEPES buffer (pH 7.40). Experiments were performed for all peptides and their copper(II) and zinc(II) complexes. ND, not determined. 14

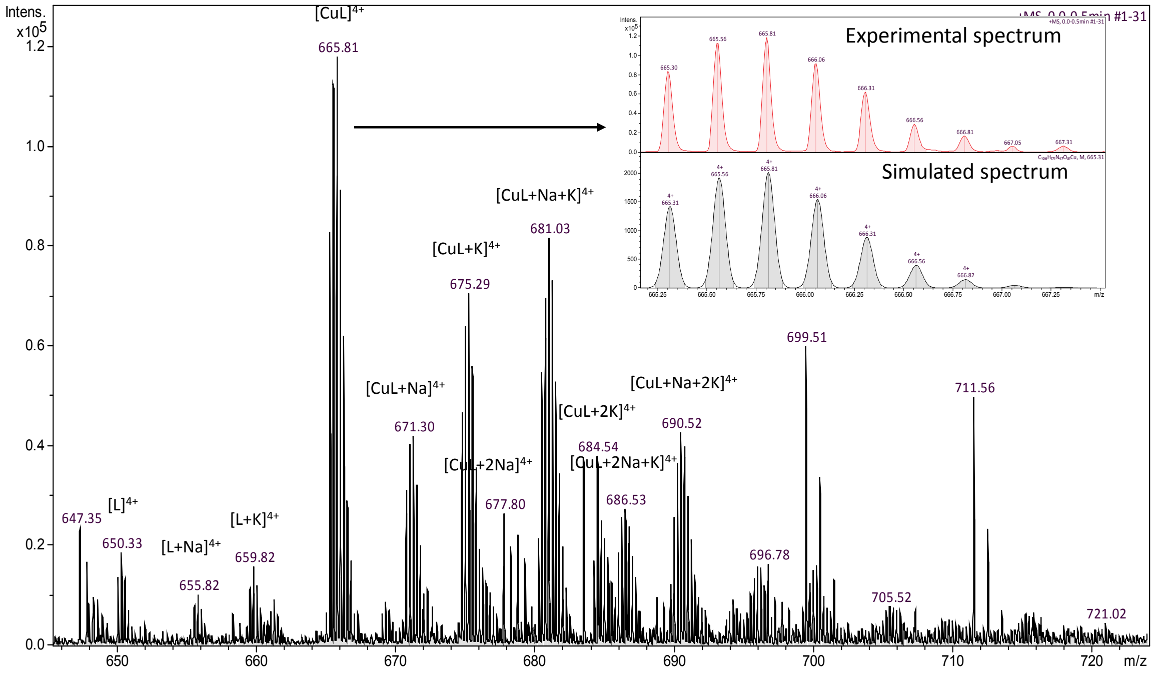

Figure S1. Mass spectra (ESI-MS) for the system Cu(II) – L1 (EGRERDHELRHRRHHHQSPK) A) whole spectrum; B) example of experimental and simulated spectrum for $[CuL]^{4+}$ complex with 1:1 stoichiometry.

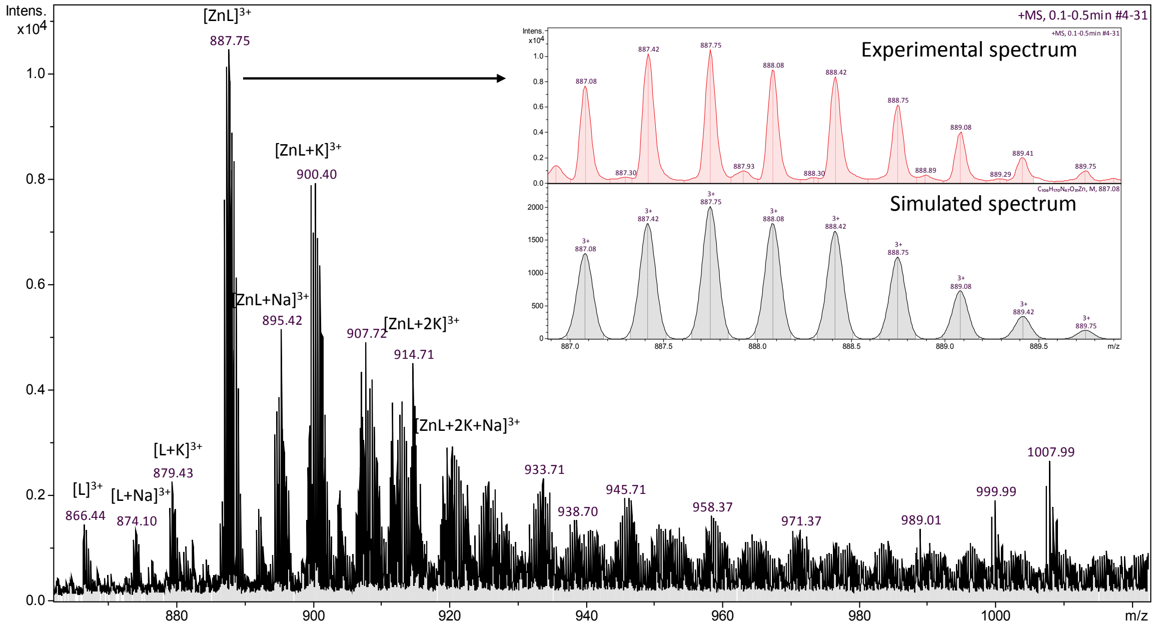

Figure S2. Mass spectra (ESI-MS) for the system Zn(II) – L1 (EGRERDHELRHRRHHHQSPK) A) whole spectrum; B) example of experimental and simulated spectrum for $[ZnL]^{3+}$ complex with 1:1 stoichiometry.

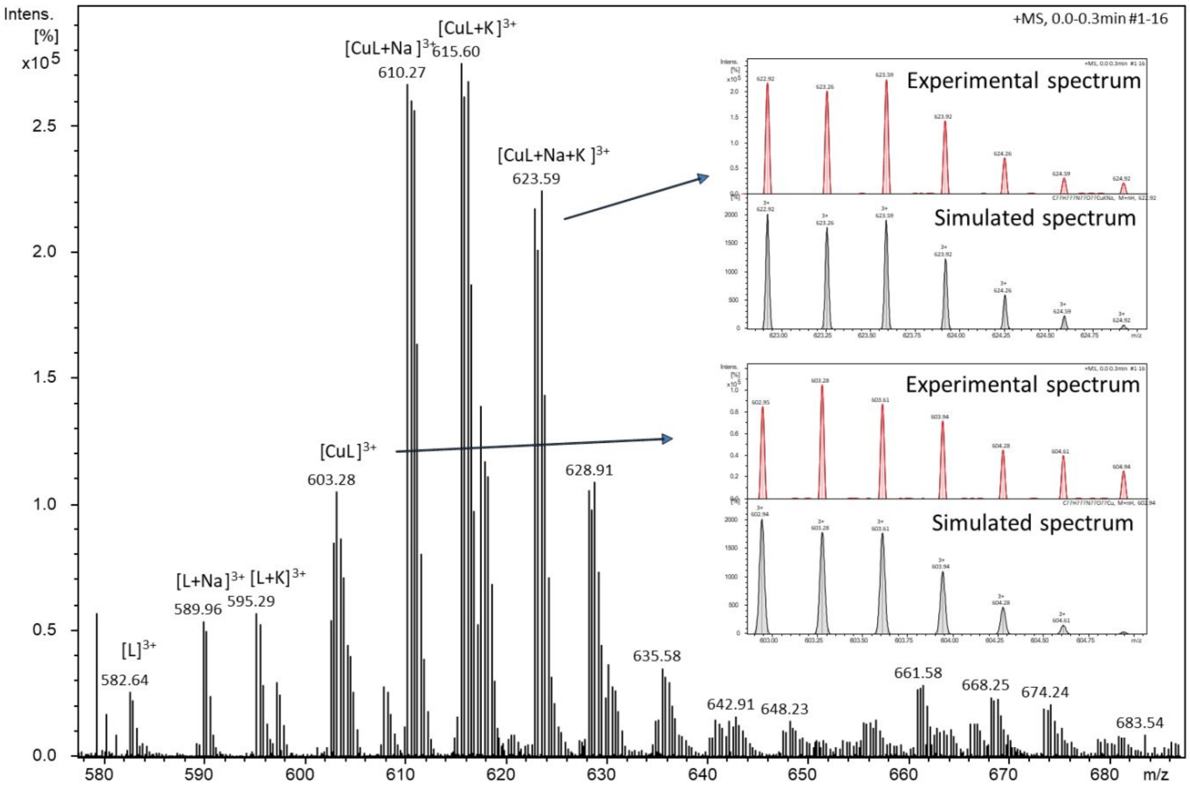

Figure S3. Mass spectra (ESI-MS) for the system Cu(II) – L2 (EGRERDHELRHRR) A) whole spectrum; B) example of experimental and simulated spectrum for $[CuL+Na+K]^{3+}$ complex; C) example of experimental and simulated spectrum for $[CuL]^{3+}$ complex with 1:1 stoichiometry.

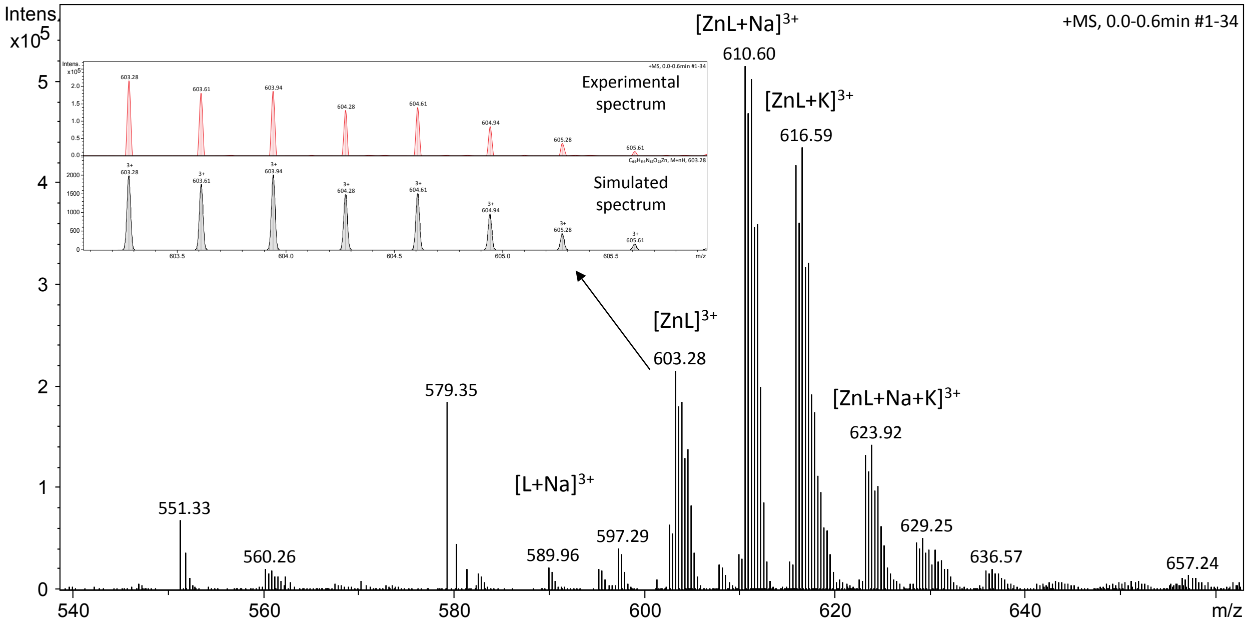

Figure S4. Mass spectra (ESI-MS) for the system Zn(II) – L2 (EGRERDHELRHRR) A) whole spectrum; B) example of experimental and simulated spectrum for $[ZnL]^{3+}$ complex with 1:1 stoichiometry.

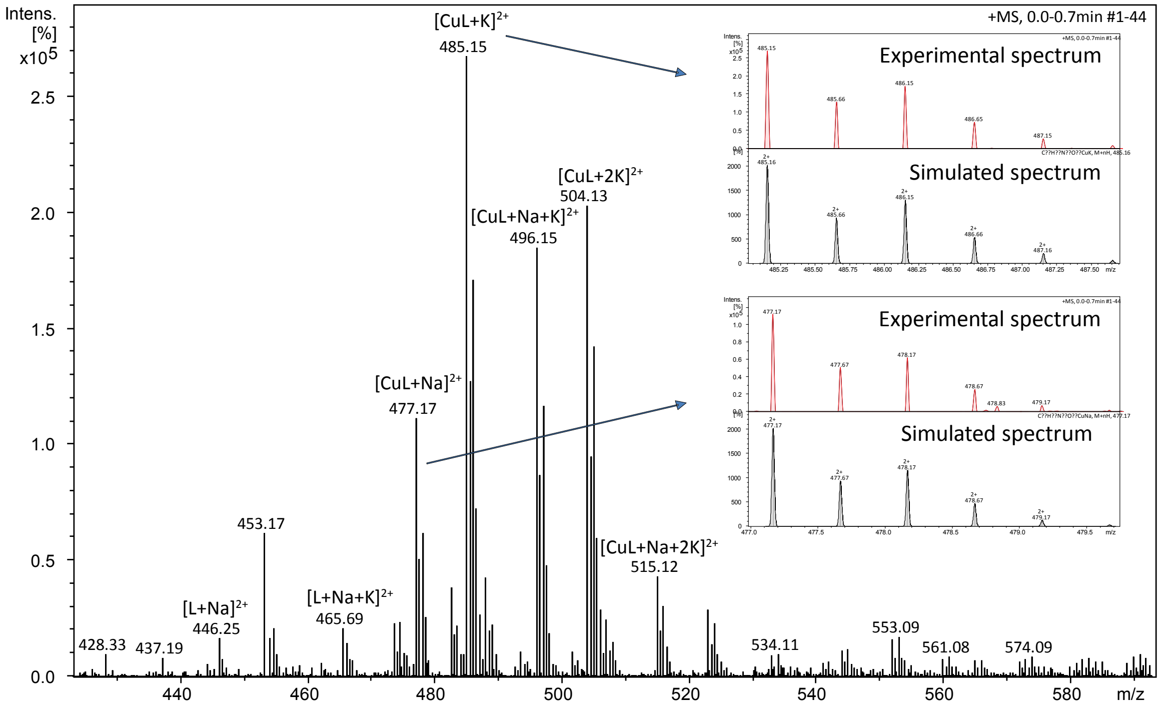

Figure S5. Mass spectra (ESI-MS) for the system Cu(II) – HHHQSPK A) whole spectrum; B) example of experimental and simulated spectrum for $[CuL+K]^{2+}$ complex; C) example of experimental and simulated spectrum for $[CuL+Na]^{2+}$ complex with 1:1 stoichiometry.

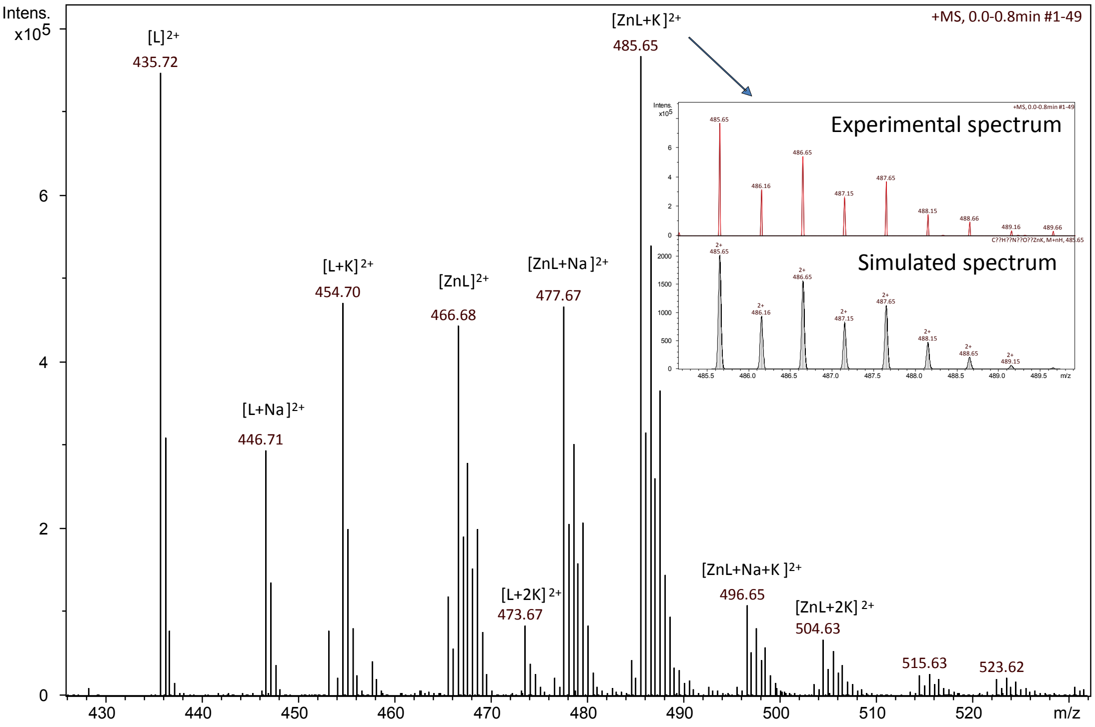

Figure S6. Mass spectra (ESI-MS) for the system Zn(II) – HHHQSPK A) whole spectrum; B) example of experimental and simulated spectrum for $[ZnL+K]^{2+}$ complex with 1:1 stoichiometry.

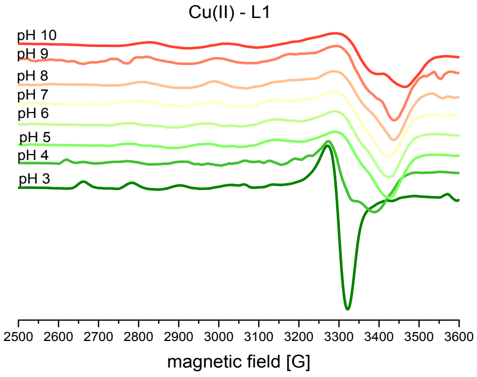

pH	$A_{\parallel}$ [G]	$g_{\parallel}$
**3**	121	2.32
**4**	136	2.30
**5**	173	2.26
**6**	177	2.25
**7**	171	2.24
**8**	183	2.23
**9**	185	2.23
**10**	195	2.21

Figure S7. pH-dependent EPR spectra for the Cu(II) - L1 (EGRERDHELRHRRHHHQSPK) system in aqueous solution with the addition of ethylene glycol (30%) at temperature 77 K. [Cu(II)] = 0.001 M, molar ratio M:L equal to 0.8:1.

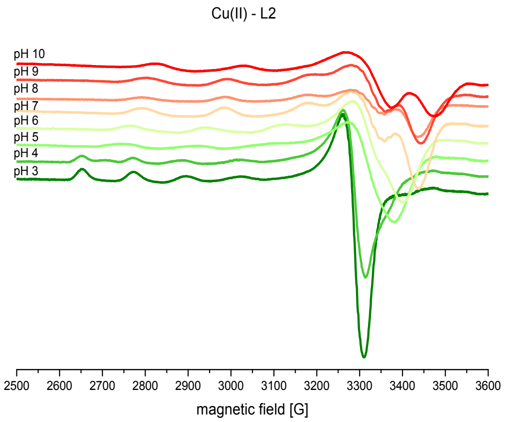

pH	$A_{\parallel}$ [G]	$g_{\parallel}$
**3**	122	2.41
**4**	139	2.43
**5**	150	2.30
**6**	180	2.27
**7**	185	2.22
**8**	195	2.22
**9**	200	2.20
**10**	206	2.19

Figure S8. pH-dependent EPR spectra for the Cu(II) – L2 (EGRERDHELRHRR) system in aqueous solution with the addition of ethylene glycol (30%) at temperature 77 K. Cu(II) concentration = 0.001 M, molar ratio M:L equal to 0.8:1.

Table S1. Thermodynamic and spectroscopic data for proton and the Cu(II) – MUC7 fragments in aqueous solution of 4 mM $HClO_4$ for each calculated complex species with the proposed coordination modes. C_L = 0.4 mM; molar ratio M:L – 0.8:1; I = 0.1 M $NaClO_4$; T = 25 °C.

	Potentiometry			***UV-Vis***		***CD***		***EPR***		***Proposed coordination mode***
Species	***logβ***	***pK_a***	***pH***	***λ[nm]***	***ε [$M^{-1}cm^{-1}$]***	***λ[nm]***	***Δε [M^{-1} cm^{-1}]***	***$A_{\parallel}$ [G]***	***$g_{\parallel}$***	
				$^{+}H_3N$-EGRERDHELLRHRRHHHQSPK-COOH (L1)						
$[CuH_6L]^{3+}$	56.02(2)	-	4.5	635	32.82	238<br>256	-3.41<br>+0.35	173	2.26	**2N**<br>{$2N_{im}$},<br>polymorphic binding states<br>Deprotonation of His (with pK_a=5.87 in the ligand)
$[CuH_5L]^{2+}$	51.20(2)	4.82	5.1	627	55.53	239	-2.80	173	2.26	**2N**

						258	+0.57			$\{2N_{im}\}$, polymorphic binding states Deprotonation of His (with pK_a=6.22 in the ligand)
$[CuH_4L]^+$	45.84(3)	5.36	6.0	616	75.85	240<br>261<br>655	-2.72<br>+0.87<br>+0.28	177	2.25	**2N** $\{2N_{im}\}$, polymorphic binding states Deprotonation of His (with pK_a =6.67 in the ligand)
$[CuH_3L]$	39.25(4)	6.59	6.8	616	93.11	243<br>261<br>343<br>651	-1.44<br>+1.94<br>-0.18<br>+0.41	171	2.24	**2N** $\{2N_{im}\}$, polymorphic binding states Deprotonation of His (with pK_a =7.44 in the ligand)
$[CuH_2L]^-$	32.20(3)	7.05	7.7	599	89.00	235<br>259<br>342<br>540<br>651	-1.11<br>+2.43<br>-0.51<br>-0.21<br>+0.35	183	2.23	**3N** $\{2N_{im}, 1N_{am}\}$ N^-_{amide} coordination
$[CuHL]^{2-}$	23.98(4)	8.22	8.7	585-542	80.64	235<br>259<br>342<br>541<br>643	-0.61<br>+3.55<br>-0.90<br>-0.26<br>+0.37	185	2.22	**3N** $\{2N_{im}, 1N_{am}\}$ and **4N** $\{2N_{im}, 2N_{am}\}$ Second N^-_{amide} coordination
$[CuL]^{3-}$	14.75(5)	9.23	9.5	525	85.05	237<br>259<br>342<br>525<br>635	-0.45<br>+4.14<br>-0.73<br>-0.44<br>+0.45	195	2.21	**4N** $\{2N_{im}, 2N_{am}\}$ Non-bonding deprotonation of N-terminus (with pK_a =9.25 in the ligand)
$[CuH_{-1}L]^{4-}$	-4.88(5)	9.87	10.1	517	132.24	235<br>263<br>350<br>506<br>637	-0.35<br>+4.71<br>-0.38<br>-0.84<br>+0.61	195	2.21	**4N** $\{1N_{im}, 3N_{am}\}$ Third N^-_{amide} coordination
$[CuH_{-3}L]^{6-}$	-15.42(4)	-	11.5	-				-	-	**4N** $\{1N_{im}, 3N_{am}\}$ Non-bonding deprotonation of Lys and Arg
				^+H_3N-EGRERDHELLRHRR-COOH (L2)						
$[CuHL]^{2-}$	13.2(1)	-	5.8	633	66.78	239	-1.85	180	2.27	**2N** $\{2N_{im}\}$

						595	-0.24			Coordination of two His (with pK_a =5.88 and 6.59 in the ligand)
$[CuL]^{3-}$	6.64(1)	6.56	7.7	609	70.53	240<br>285<br>597	-1.93<br>-0.36<br>-0.34	195	2.22	**3N**<br>{$2N_{im}$, $1NH_2$}<br>Coordination of N-terminus (with pK_a =7.56 in the ligand)
$[CuH_{-1}L]^{4-}$	-2.20(1)	8.84	8.9	560	77.44	546<br>298<br>236<br>256	-0.34<br>+0.06<br>-1.70<br>1.07	200	2.20	**4N**<br>{$2N_{im}$, $1NH_2$, $1N_{am}$}<br>Coordination of first N^{-}_{amide}
$[CuH_{-2}L]^{5-}$	-11.17(1)	8.97	9.5	531	95.12	237<br>257<br>284<br>343<br>547	-1.83<br>+1.67<br>-0.20<br>-0.19<br>-0.47	206	2.19	**4N**<br>{$1N_{im}$, $2N_{am}$, $1NH_2$}<br>Coordination of second N^{-}_{amide}
$[CuH_{-3}L]^{6-}$	-21.06(1)	9.89	11.9	517	122.92	236<br>255<br>284<br>545	-2.35<br>+1.75<br>-0.70<br>-0.77	-	-	**4N**<br>{$1N_{im}$, $3N_{am}$}<br>or<br>{$3N_{am}$, $1NH_2$}<br>Coordination of third N^{-}_{amide}
					$^{+}H_3N$-HHHQSPK-COOH (L3)					
$[CuH_3L]^{4+}$	31.81	-	3.4	600	42.13	236<br>269<br>298<br>616	-2.81<br>+0.03<br>-0.41<br>+0.05	-	-	**2N**<br>{$1N_{im}$, $1NH_2$}<br>Coordination of His (with pK_a =4.88 in the ligand) and N-terminus (with pK_a =7.49 in the ligand)
$[CuH_2L]^{3+}$	28.62	3.19	4.0	583	51.28	237<br>268<br>296<br>617	-2.82<br>+0.02<br>-0.44<br>+0.126	-	-	**3N**<br>{$2N_{im}$, $1NH_2$}<br>Coordination of His (with pK_a =5.84 in the ligand)
$[CuHL]^{2+}$	24.12	4.50	5.3	528	97.81	239<br>274<br>315<br>485<br>572	-1.59<br>-2.82<br>+0.48<br>+0.52<br>-0.53	-	-	**4N**<br>{$2N_{im}$, $1NH_2$, $1N_{am}$}<br>Coordination of first N^{-}_{amide}
$[CuL]^{+}$	18.13	5.99	6.7	527	110.91	244<br>274<br>315	-1.45<br>-3.46<br>+0.49	-	-	**4N**<br>{$2N_{im}$, $1NH_2$, $1N_{am}$}<br>Non-bonding Deprotonation of His (with pK_a =6.52 in the ligand)

						485	+0.69			
						573	-0.49			
[CuH$_{-1}$L]	10.64	7.49	8.9	524	121.02	243	-0.93	-	-	**4N** {1N$_{im,}$ 1NH$_{2,}$ 2N$_{am}$} Coordination of second N$^{-}_{amide}$
						275	-3.79			
						319	+0.69			
						486	+0.78			
						566	-0.61			
[CuH$_{-2}$L]$^{-}$	0.32	10.32	> 12.0	525	130.79	276	-3.63	-	-	**4N** {1N$_{im,}$ 1NH$_{2,}$ 2N$_{amid}$} Non-bonding Deprotonation of Lys with pK_a =10.33 in the ligand)
						318	+0.83			
						486	+0.75			
						566	-0.65			

Table S2. Thermodynamic data for proton and the Zn(II) – MUC7 fragments in aqueous solution of 4 mM $HClO_4$ for each calculated complex species with the proposed coordination modes. C_L = 0.4 mM; molar ratio M:L – 0.8:1; I = 0.1 M $NaClO_4$; T = 25 ^{o}C.

Species	***logβ***	***pK$_a$***	***pH***	***Proposed coordination mode***
		$^{+}$H$_3$N-EGRERDHELLRHRRHHHQSPK-COOH (L1)		
[ZnH$_5$L]$^{2+}$	48.31(2)	-	5.8	**3N** {3N$_{im}$} polymorphic binding sites Deprotonation of His (with pK_a =6.22 in the ligand)
[ZnH$_4$L]$^{+}$	42.25(2)	6.06	6.3	**3N** {3N$_{im}$}, polymorphic binding sites Deprotonation of His (with pK_a =6.67 in the ligand)
[ZnH$_3$L]	35.69(2)	6.56	7.3	**3N** {3N$_{im}$}, polymorphic binding sites Deprotonation of His (with pK_a =7.44 in the ligand)
[ZnH$_2$L]$^{-}$	27.8(3)	7.89	8.4	**4N** {3N$_{im,}$ 1NH$_2$} Deprotonation of N-terminal (with pK_a =9.25 in the ligand)
[ZnHL]$^{2-}$	18.93(4)	8.87	9.3	**4N** {3N$_{im,}$ 1NH$_{2,}$ OH^{-}} Deprotonation of aqua ligand

[ZnL]$^{3-}$	9.25(5)	9.68	9.9	**4N** {3N$_{im,}$ 1NH$_{2,}$ 2 OH^{-}} Deprotonation of aqua ligand
[ZnH$_{-2}$L]$^{5-}$	-10.97(5)	-	11.2	**4N** {3N$_{im,}$ 1NH$_{2,}$ 2 OH^{-}} Deprotonation of Lys and Arg

$^{+}H_3$N-EGRERDHELLRHRR-COOH (L2)

[ZnHL]$^{2-}$	11.31(3)	-	6.5	**2N** {2N$_{im}$} Deprotonation of His (with pK_a =6.59 in the ligand)
[ZnL]$^{3-}$	4.43(2)	6.88	7.8	**3N** {2N$_{im}$, 1NH$_2$} Deprotonation of N-terminal (with pK_a =7.56 in the ligand)
[ZnH$_{-1}$L]$^{4-}$	-4.18(4)	8.61	9.1	**3N** {2N$_{im}$, 1NH$_2$, 1 OH^{-}} Deprotonation of aqua ligand

$^{+}H_3$N-HHHQSPK-COOH (L3)

[ZnHL]$^{2+}$	15.94(2)	-	6.4	**4N** {3N$_{im}$, 1NH$_2$} or **3N** {2N$_{im}$, 1NH$_2$} Deprotonation of His (with pK_a =6.52 in the ligand) or N-terminal (with pK_a =7.49 in the ligand)
[ZnL]$^{+}$	9.02(3)	6.92	8.3	**3N** {2N$_{im}$, 1NH$_2$, 1 OH^{-}} Deprotonation of aqua ligand
[ZnH$_{-1}$L]	-0.71(4)	9.73	10.1	**3N** {2N$_{im}$, 1NH$_2$, 2 OH^{-}} Deprotonation of aqua ligand
[ZnH$_{-2}$L]$^{-1}$	-11.2(4)	10.49	11.9	**3N** {2N$_{im}$, 1NH$_2$, 2 OH^{-}} Deprotonation of Lys (with pK_a =10.33 in the ligand)

Table S3. *In vitro* antibacterial activity of peptides/complexes determined as a minimal inhibitory concentration (MIC) (µg/mL); antimicrobial assays were performed in 10 mM MES buffer (pH 5.40). Experiments were performed for all peptides and their copper(II) and zinc(II) complexes. ND, not determined.

**pH 5.40**	*E. coli* ATCC 25922	*P. aeruginosa* ATCC 15422	*E. faecalis* ATCC 29212	*S. aureus* ATCC 25923	*S. mutans* PMC 2502	*S. sanguinis* PMC 2335	*C. albicans* SC5314
**L1**	n/d	n/d	n/d	n/d	n/d	n/d	n/d
**Cu(II) – L1**	n/d	n/d	n/d	n/d	n/d	n/d	n/d
**Zn(II) – L1**	n/d	n/d	n/d	n/d	n/d	n/d	n/d
**L2**	n/d	n/d	n/d	n/d	n/d	n/d	n/d
**Cu(II) – L2**	n/d	n/d	n/d	n/d	n/d	**250**	n/d
**Zn(II) – L2**	n/d	n/d	n/d	n/d	n/d	**250**	n/d
**L3**	n/d	n/d	n/d	n/d	n/d	**250**	n/d
**Cu(II) – L3**	n/d	n/d	n/d	n/d	n/d	**250**	n/d
**Zn(II) – L3**	n/d	n/d	n/d	n/d	n/d	**125**	n/d
**Cu(II)**	n/d	n/d	n/d	n/d	n/d	n/d	n/d
**Zn(II)**	n/d	n/d	n/d	n/d	n/d	n/d	n/d

Table S4. *In vitro* antibacterial activity of peptides/complexes determined as a minimal inhibitory concentration (MIC) (µg/mL); antimicrobial assays were performed in 10 mM HEPES buffer (pH 7.40). Experiments were performed for all peptides and their copper(II) and zinc(II) complexes. ND, not determined.

**pH 7.40**	*E. coli* ATCC 25922	*P. aeruginosa* ATCC 15422	*E. faecalis* ATCC 29212	*S. aureus* ATCC 25923	*S. mutans* PMC 2502	*S. sanguinis* PMC 2335	*C. albicans* SC5314
**L1**	n/d	n/d	n/d	n/d	n/d	n/d	n/d
**Cu(II) – L1**	n/d	n/d	n/d	n/d	n/d	n/d	n/d
**Zn(II) – L1**	n/d	n/d	n/d	n/d	n/d	**500**	n/d
**L2**	n/d	n/d	n/d	n/d	n/d	n/d	n/d
**Cu(II) – L2**	n/d	n/d	n/d	n/d	n/d	n/d	n/d
**Zn(II) – L2**	n/d	n/d	n/d	n/d	n/d	**500**	n/d
**L3**	n/d	n/d	n/d	n/d	n/d	**500**	n/d
**Cu(II) – L3**	n/d	n/d	n/d	n/d	n/d	n/d	n/d
**Zn(II) – L3**	n/d	n/d	n/d	n/d	n/d	**250**	n/d
**Cu(II)**	n/d	n/d	n/d	n/d	n/d	n/d	n/d
**Zn(II)**	n/d	n/d	n/d	n/d	n/d	n/d	n/d
